# Preparation and characterization of supported magnetic nanoparticles prepared by reverse micelles

**DOI:** 10.3762/bjnano.1.5

**Published:** 2010-11-22

**Authors:** Ulf Wiedwald, Luyang Han, Johannes Biskupek, Ute Kaiser, Paul Ziemann

**Affiliations:** 1 Institut für Festkörperphysik, Universität Ulm, 89069 Ulm, Germany; 2 Materialwissenschaftliche Elektronenmikroskopie, Universität Ulm, 89069 Ulm, Germany

**Keywords:** Co, CoPt, core–shell particles, FePt, magnetic anisotropy, magnetic particles, plasma etching, reverse micelles, self-assembly

## Abstract

Monatomic (Fe, Co) and bimetallic (FePt and CoPt) nanoparticles were prepared by exploiting the self-organization of precursor loaded reverse micelles. Achievements and limitations of the preparation approach are critically discussed. We show that self-assembled metallic nanoparticles can be prepared with diameters *d* = 2–12 nm and interparticle distances *D* = 20–140 nm on various substrates. Structural, electronic and magnetic properties of the particle arrays were characterized by several techniques to give a comprehensive view of the high quality of the method. For Co nanoparticles, it is demonstrated that magnetostatic interactions can be neglected for distances which are at least 6 times larger than the particle diameter. Focus is placed on FePt alloy nanoparticles which show a huge magnetic anisotropy in the L1_0_ phase, however, this is still less by a factor of 3–4 when compared to the anisotropy of the bulk counterpart. A similar observation was also found for CoPt nanoparticles (NPs). These results are related to imperfect crystal structures as revealed by HRTEM as well as to compositional distributions of the prepared particles. Interestingly, the results demonstrate that the averaged effective magnetic anisotropy of FePt nanoparticles does not strongly depend on size. Consequently, magnetization stability should scale linearly with the volume of the NPs and give rise to a critical value for stability at ambient temperature. Indeed, for diameters above 6 nm such stability is observed for the current FePt and CoPt NPs. Finally, the long-term conservation of nanoparticles by Au photoseeding is presented.

## Introduction

Magnetic nanoparticles have been the focus of research for over 60 years [1–2]. These investigations were prompted by both, fundamental aspects of the magnetism of small particles and clusters [3–4], and an increasing interest by industry, especially in the field of data storage (first magnetic tapes and later hard disk drives) [5–6]. Other important current applications include their use in the medical field [7], e.g., in hyperthermia [8], contrast enhancing in magnetic resonance imaging [9–10] or the use as cell markers [9] which in-turn can be read out by highly-sensitive devices like TMR-sensors [11]. Moreover, magnetic NPs are thought to improve a variety of catalytic reactions [12–13]. Note that in this case the magnetic properties are in the background, rather fine tuning electronic properties of metallic surfaces is in the principle focus directed towards the catalytic activity of the NPs [14].

Various methods have been used to prepare magnetic NPs with diameters of 1–30 nm. Possibly the simplest approach is ball milling of the corresponding bulk materials. This mostly yields a rather broad size distribution, which, in turn, often hinders the study of size-dependent properties [15]. A better defined physical approach is inert-gas condensation where NPs are formed by sputtering atoms from a specific target which then agglomerate into clusters in a continuous gas flow before landing on a support [16–17]. This method has the advantage of full processing under vacuum conditions and, moreover, monodisperse NPs can be prepared by size selection during flight [18–19]. One general drawback is the random deposition of NPs on the substrate. When properties of individual NPs are of interest, then only low coverage of NPs is necessary before agglomerates are formed on the support. Note that by landing on a polymer matrix [20] or alternatively, on a biotemplate [21] it is possible to avoid the latter drawback to some extent.

The impressive progress in organometallic chemistry, however, has revolutionized the field of small particles for more than a decade [22–23]. Surfactant-mediated growth [24] of NPs with narrow size distributions from metal precursors in solution opened the field of self-assembly, which allows the formation of large-scale ordered arrays of NPs on a support [25–27]. Over the years this method has been optimized by many groups to prepare NPs with tunable diameters, small size distributions with small nm interparticle spacings and additionally, the flexibility to produce monatomic [28] as well as bimetallic NPs [24,29–30]. However, due to the preparation technique the NPs are covered by surfactants which may alter the magnetic properties of NPs [31].

Common to all the mentioned approaches is that oxides are formed when the NPs are exposed to ambient conditions. Thus, much effort has been spent on the removal of organic cover layers leading to naked particles on a support and subsequent reduction of NPs to yield, ultimately, purely metallic species [32–33]. It was shown that subsequent processing by oxygen and hydrogen plasmas is the key to obtain individual metallic particles [34]. On the other hand, embedding the NPs in an antiferromagnetic matrix may lead to modified magnetic properties due to exchange bias giving rise to thermal stability at ambient temperature with NPs having intrinsically low anisotropies [35].

Parallel to the so-called colloidal approach where NPs are formed within a liquid, the preparation of precursor loaded reverse micelles has been developed [36–37]. Here, precursor filled di*block*-co-polymers are used to form hexagonally ordered arrays on different substrates by dip-coating [38]. In a second step, NPs are formed on the substrates by exposure to oxygen plasma which etches the polymers and simultaneously forms metal-oxide NPs [39]. On subsequent hydrogen plasma treatment the formed NPs can be converted into their metallic state. This approach succeeded in the preparation of monatomic Au [40], Pt [41], Fe, Co NPs as well as bimetallic NPs such as FePt, CoPt [42] (and this present contribution) to mention only a few. Unlike all other preparation methods, the micelle approach leads to supported NPs with significantly larger interparticle distances (*D* > 20 nm) which is especially appealing for two reasons: (i) the magnetostatic interaction of NPs is very small and consequently, individual magnetic properties can by extracted, and (ii) the larger distance between NPs allows their annealing as opposed to colloidal NPs where extended heat treatment leads to agglomeration [43]. This aspect is especially useful for systems which undergo a phase transition and thereby improves their magnetic properties such as in the cases of FePt or CoPt NPs which are of technological interest due to their high magnetocrystalline anisotropy in the L1_0_ phase. However, it has mostly been observed that the as-prepared particles exhibit the chemically disordered A1 (fcc) phase, while annealing at typically 600–700 °C the NPs are partially transformed into the chemically ordered L1_0_ phase [23].

Besides the large interparticle distance, the preparation route presented in this contribution allows the systematic variation of particle diameters with narrow size distributions [44]. Thus, possible size-effects [45], e. g., of the effective magnetic anisotropy, can be investigated. In the literature, the achieved hard magnetic properties of FePt NPs and thin films after annealing vary widely [46], since the structure [47], chemical composition [48–49] and the degree of chemical order [50] have to be optimized. Ideally, each particle should be single-crystalline at equiatomic composition and perfectly L1_0_ ordered. Meeting these three premises, however, is difficult.

One remarkable concept is the salt-annealing technique [51] in which colloidal particles are dispersed at low concentration in a salt matrix which allows high-temperature annealing without NP coalescence. After removal of the salt matrix and recovery of NPs applying surfactants as spacers, the particles show size-dependent degrees of chemical order, coercive fields, saturation magnetizations, and Curie temperatures. Technologically important is the report of the last noted group, that after annealing of FePt NPs as small as 4 nm, ferromagnetic behavior is observed even at ambient temperature corresponding to magnetic anisotropies close to those of the bulk material [52]. To the best of our knowledge, this result has, so far, not been reproduced by other groups. In contrast, most reports find reduced anisotropies in L1_0_ ordered NPs compared to their bulk counterpart [53], which mostly is ascribed to surface effects or defects and twins in the NP structure [47]. Consequently, a systematic investigation of possible size-dependent properties of FePt NPs is highly desirable and may lead to further insights on the ordering process. Such an investigation requires, however, a reproducible route towards ensembles of FePt NPs with as narrow as possible diameter distributions. The presently introduced micellar route, which offers control of both particle size and interparticle distance, fulfills this requirement to a considerable extent.

## Results and Discussion

In this article we address the preparation of magnetic NPs by precursor loaded reverse micelles on different supports (section 1). The formation of metallic NPs by means of plasma etching was investigated in more detail by X-ray photoelectron spectroscopy as described in section 2. Moreover, the structure of FePt alloy NPs was determined by high resolution transmission electron microscopy and their tendency for Pt segregation in the metallic state by annealing as well as their stability in ambient conditions are discussed. Section 3 focuses on the magnetic properties of Co, CoPt and FePt NPs. We show that magnetostatic interactions can be neglected for micelle-based NPs, which is used in a step where the effective anisotropy *K*_eff_ of FePt and CoPt is determined by simple Stoner**–**Wohlfarth approach using a bimodal distribution of *K*_eff_. Finally in section 4, the first results on Au photoseeding of Co NPs are presented.

### 1. Preparation of supported nanoparticles

#### 1.1 General preparation route based on micelles

As already mentioned in the introduction, a vast variety of preparation approaches have been developed and more or less successfully tested for their application to fabricate ferromagnetic metal NPs. Success, in this context, may not be an appropriate term, since it critically hinges on the specific requirements on the NPs, which, in turn, depend on the particular application. For example, magnetic data storage using NPs demands non-interacting particles which immediately translates into a lower limit of the interparticle distances. Furthermore, retrieving the stored information requires well-defined spatial particle arrangements, for example periodic NP arrays. Yet another application driven requirement might be to keep the variance of magnetic properties within a given particle ensemble within a pre-defined narrow range. This again can be translated into corresponding requirements on size and shape as well as the chemical composition of the magnetic NPs.

Thus, before giving a success judgment of the following preparation route based on the self-organization of micelles, the various desired criteria for magnetic particles are listed below:

- Homogeneously shaped NPs (e. g., spheres)
- Narrow size distributions (throughout this article, size will be expressed by an average diameter)
- Spatially ordered arrays of NPs, in the ideal case a 2-dimensional periodic lattice of NPs.

In addition to these NP related requirements, any fabrication process should offer high versatility with respect to the desired species of NPs such as various elemental or alloyed systems as well as control of pre-defined NP size and interparticle distance. Finally, since in the following NPs are addressed which are exclusively supported on a substrate, the preparation process should be applicable to supports with different chemical properties.

**Scheme 1:**
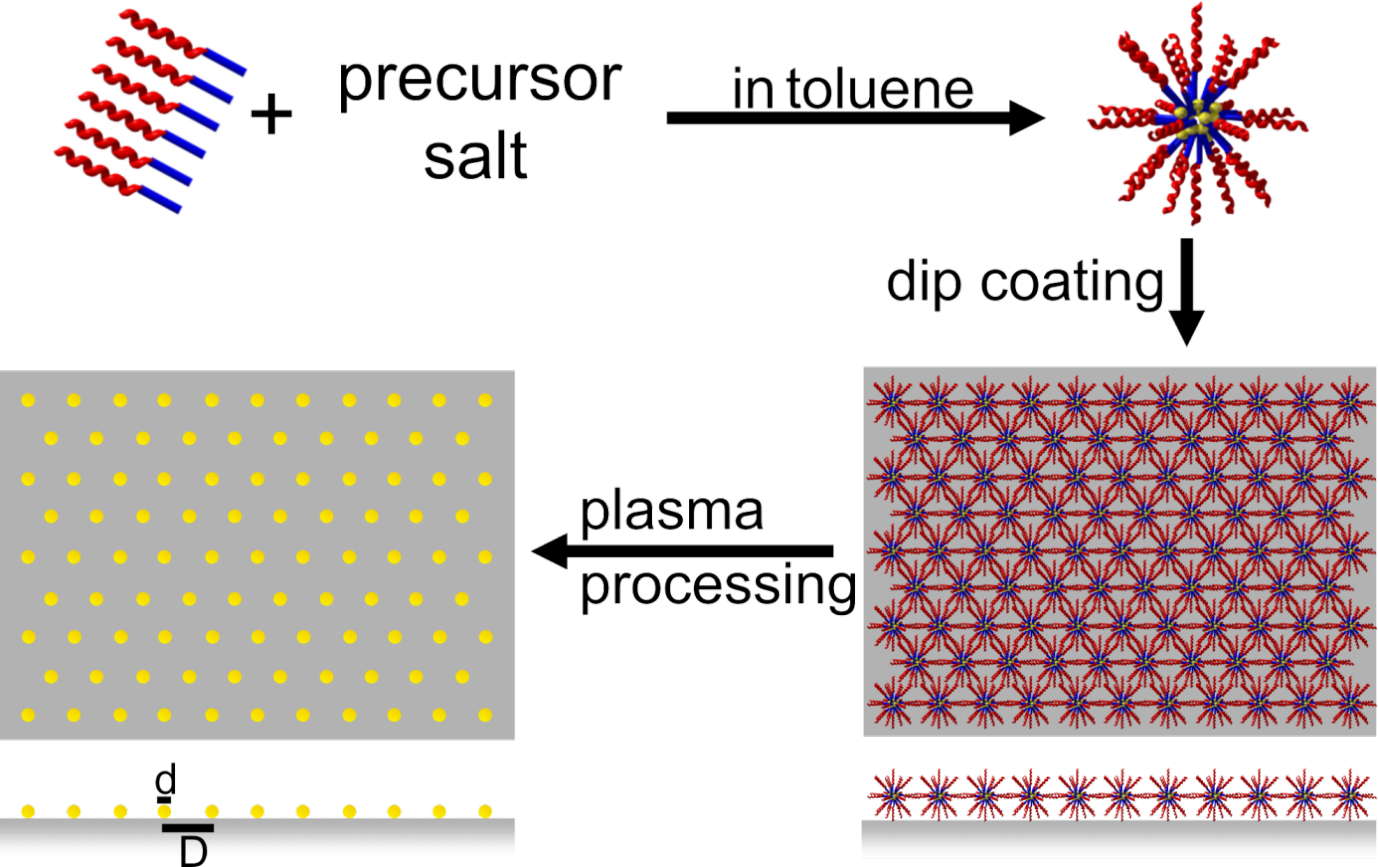
Preparation of NPs by a reverse micelle technique. PS-*b*-P2VP or PS-*b*-P4VP is dispersed in anhydrous toluene. After adding a metal precursor salt and continuous stirring for about one week, reverse micelles are homogeneously loaded with the precursor material. By dip coating a hexagonal arrangement of loaded micelles can be obtained. This micelle monolayer is exposed successively to oxygen and hydrogen plasmas for (i) removal of the polymer and particle nucleation and (ii) the reduction of the metal oxide particles, respectively.

The basic idea of how to approach all those aims given above may be addressed as “carrier-principle”: A macromolecular carrier is sought which can be prepared in a liquid solvent and which exhibits a genuine tendency towards self-organization into an ordered array on top of a given substrate. In the case of spherical carriers that tend to close packing, after evaporation of the solvent a supported hexagonal arrangement may be expected. In addition, the carrier has to provide a loading mechanism to allow the chemically bonded precursor material to penetrate its interior. With those two prerequisites in mind, a suitable precursor for the planned species of NPs is loaded into the carriers during their formation or presence in the solution and, subsequently, is transported by them to the ordered positions defined by the self-organization of the carrier. In the next, most delicate step of the fabrication procedure, the organic carriers are completely removed by a plasma process and, simultaneously, the loaded precursor material is transformed into NPs of the desired material whilst conserving their original carrier position. In this way, the order of the self-organized carrier array is mapped onto the finally obtained spatial arrangement of the NPs. This basic idea of preparation and the different steps involved are summarized in the schematics shown in Scheme 1.

The first experimental realization of the above approach was demonstrated by the preparation of hexagonally ordered arrays of Au NPs [54]. In this case inverse spherical micelles formed from di*block*-co-polymers in anhydrous toluene were used as carriers. It was shown that polystyrene-*block*-poly(2-vinylpyridine) (PS-*b*-P2VP) or polystyrene-*block*-poly(4-vinylpyridine) (PS-*b*-P4VP) di*block*-co-polymers with the hydrophilic P2VP block formed the core of the micelles in the apolar solvent and the hydrophobic PS block the surrounding shell. Loading of precursor material is selectively provided by the pyridine groups of the core. This micellar approach has been continuously improved over recent years [55–56] and is the focus of the present contribution. It is worth noting, however, that in the interim the carrier principle has also been transferred to spherical colloidal polystyrene (PS) particles which can be loaded with various metal precursors by emulsion and miniemulsion methods [57–58].

#### 1.2 Polymers and precursors

In the following we will concentrate exclusively on the fabrication of ordered arrays of magnetic NPs based on the self-organization of precursor-loaded micelles. Application of this approach to obtain magnetic NPs has been previously reported [34]. Continuous optimization and simplification of the process, however, including replacement of the ‘home-made’ [54] co-polymers by commercially available ones, suggest it might be possible to obtain some supplementary information on the present state-of-the-art. For this purpose, we follow the sequence of the micellar process steps and start with the presently used polymer and precursor materials.

Commercially available di*block*-co-polymers were used exclusively (Polymer Source Inc., Canada) of the type PS(x)-*b*-P2VP(y) or PS(x)-*b*-P4VP(y), where x and y denote the number of monomers per block and, thus, determine the length of each block. Due to the hydrophobicity of the PS- and hydrophilicity of PVP-block, the co-polymers form reverse spherical micelles in apolar solvents such as the solvent used here, i.e., toluene. Empirically, however, in order to obtain stable spherical micelles, the block length of PS, x, should be significantly larger than the PVP length y. In practice, when x ≥ 2y, the optimized spherical micelles are obtained.

**Figure 1:**
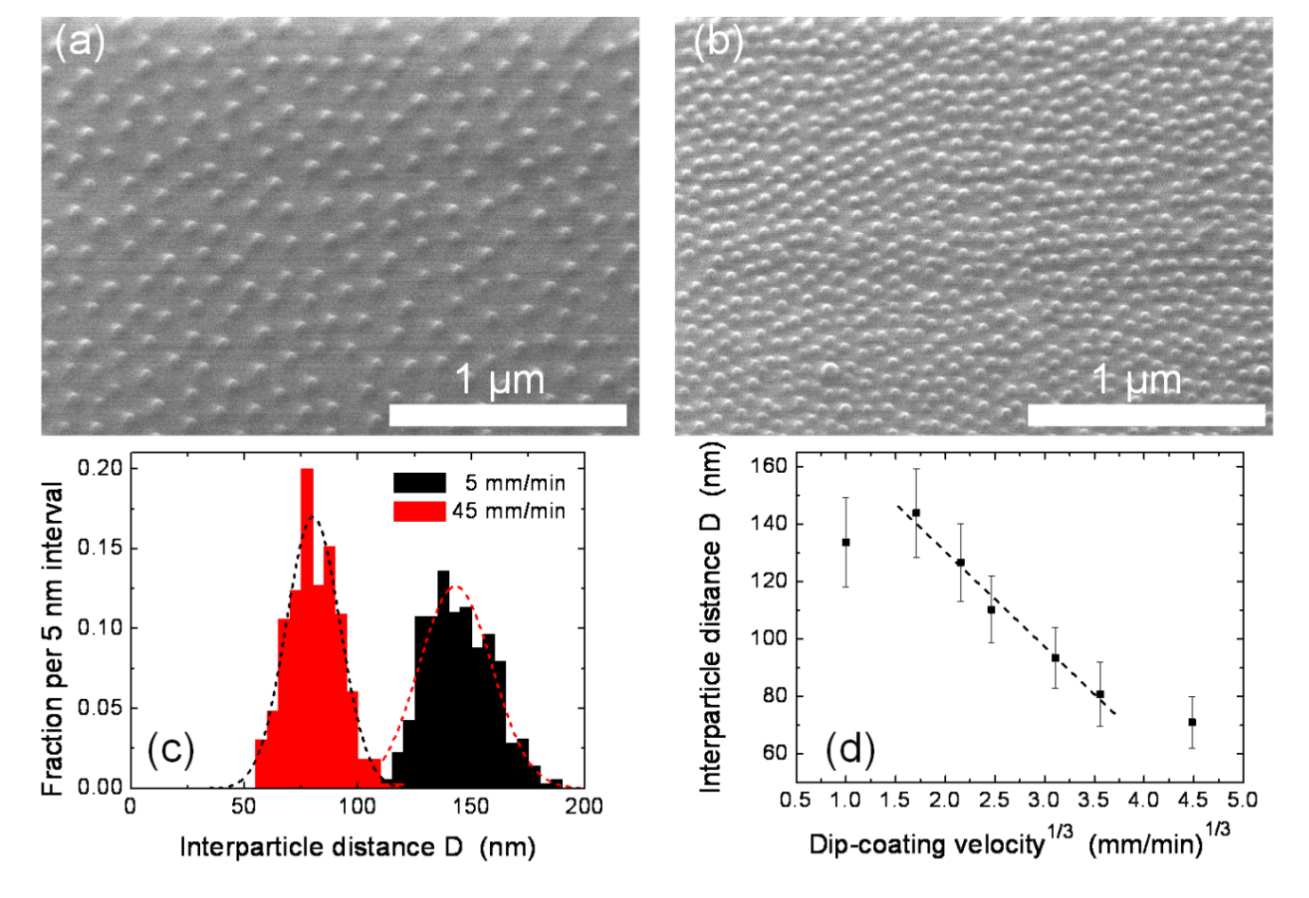
CoCl_2_ loaded PS(1779)-b-P2VP(857) reverse micelles deposited at different dip-coating velocities; (a) and (b) show the SEM micrographs at dip-coating velocities of 5 mm/min and 45 mm/min, respectively. The corresponding distributions of the interparticle distance D and Gaussian fits are shown in (c). Panel (d) shows the interparticle distance as function of the 3^rd^ root of the dip-coating velocity in the range of 1–90 mm/min. The dashed line is a linear fitting of the five central points.

On the other hand, the parameter y indicates the number of pyridine moieties per co-polymer which serve as binding sites for the precursor units. In the simplest approximation, the number of pyridine sites per micelle scales linearly with the volume of the finally expected NPs. Furthermore, the center-to-center distance of the spherical micelles should be proportional to (x + y) which parameterizes the total length of the co-polymer strand. From all the above, it becomes clear that the final size *d* of the NPs is not completely independent of the interparticle distance *D* (cf. Figure 1). Assuming *D* ≈ 2(x + y) and x ≥ 2y, one finds the estimate y ≤ *D*/6. If y^3^ is proportional to the final volume *V*, it follows directly that y is proportional to the nanoparticle diameter *d*. Thus, an estimate of the correlation of the particle diameter *d* and the interparticle distance *D* may be given by 6*d* ≤ *D*. The validation of this relation is shown in the next section.

Once a suitable di*block*-co-polymer has been chosen, the corresponding solution is prepared by stirring the polymer in toluene for typically one week at ambient temperature. This period can be shortened to overnight stirring by increasing the temperature to 50 °C. In this case, however, the long-time stability of the micelles appears to be significantly reduced. Some additional practical caveats may be worth mentioning:

- It appears good practice to re-check the length distributions of the co-polymers, e.g., by size-exclusion chromatography (SEC) to make sure there is only one dominating peak.
- If toluene is used as solvent, care should be taken to keep it anhydrous.
- Most of the metal precursor salts are sensitive to humidity and, consequently, exposure to ambient conditions should be avoided or at least minimized.

Despite all of the above, the use of a glove box did not prove necessary for obtaining optimized NPs.

The other important components for the micellar recipe, besides the co-polymers forming the carriers, are the various precursor materials. This issue will be addressed by distinguishing between elemental and bi-metallic magnetic NPs.

Elemental NPs: Here our focus is on Fe and Co NPs and the related standard precursors are FeCl_3_, and CoCl_2_, respectively (purities 99.99% and 99.999% as given by Alfa Aesar). In all cases, loading of the micelles is accomplished by adding the precursor to the micellar solution and stirring for a couple of days until no more metal salt residues are visible. Homogenous loading of micelles is observed up to precursor concentrations of 0.5 precursor units per P2VP monomer unit. Above this limit, it was observed that not all of the precursor salt is solved to the cores of the micelles.

Bimetallic NPs: Here the situation is more complicated, since besides the right choice of the individual metal precursors and their amount, the sequence of their addition to the micelle solution plays a crucial role. For instance, for FePt NPs the Pt-precursors H_2_PtCl_6_, PtCl_4_ and K[PtCl_3_C_2_H_4_]∙H_2_O (‘Zeise salt’) were investigated as well as the Fe-precursors FeCl_2_ and FeCl_3_. It transpired that, on the basis of the criteria of homogeneity and completeness of loading the micelles, the combination of Zeise salt and FeCl_3_ gave the optimum results. To obtain optimized FePt NPs, however, the Pt-precursor must be added first to the micelle solution, stirred until loading is complete and only then the addition of FeCl_3_ should be started and stirred continuously until no salt residues are visible. Similarly, for the preparation of CoPt NPs the precursor sequence, Zeise salt first followed by CoCl_2_, gave the best results. Precursor salts were adjusted to the targeted 1:1 composition of the final NPs keeping the upper total loading limit of 0.5 precursor units per P2VP monomer.

#### 1.3 Deposition of loaded micelles onto various substrates

The magnetic NPs, which are the focus of the present work, have been deposited onto various, mostly planar, substrates. Standard combinations are Co and Fe NPs on Si/SiO_2_ or Pt as well as FePt on MgO, Si and sapphire. The latter substrates were single crystalline materials with (001) and (0001) orientations, respectively, while, in case of Pt, (111)-textured thin films (50 nm) were used which were obtained by pulsed laser deposition (PLD) at ambient temperature on MgO(001) or (100)-oriented films (80 nm) epitaxially grown by PLD on (001) strontium titanate (SrTiO_3_) crystals at 400 °C. In all cases, no special pre-treatment of these substrates was employed prior to the deposition of the loaded micelles.

The deposition itself was made by dip-coating. For this purpose, the substrate was immersed into the micelle solution and then pulled out under ambient conditions in a controlled way at a fixed velocity. This velocity is a critical parameter as it systematically influences the inter-micelle distance on the substrate [55]. The effect was demonstrated on CoCl_2_-loaded micelles (polymer type: PS(1779)-*b*-P2VP(857)) deposited onto Si/SiO_2_ substrates by using a variety of dip-coating velocities in the range 1–90 mm/min. The corresponding results are presented in Figure 1, where the two top panels show scanning electron microscopy (SEM) images of identical micelles deposited at different velocities of (a) 5 mm/min and (b) 45 mm/min, respectively. The corresponding different areal densities of the micelles are clearly visible. Analyzing the SEM images in more detail shows the related distributions of the inter-micelle distances (Figure 1(c)), which can be approximated by Gaussian distributions (dashed curves). From such distributions the average distances are determined and plotted against the 3^rd^ root of the dip-coating velocity. Similar to previous results with Au loaded micelles [55,59], within a relatively broad range of velocities a linear relationship is obtained in accord with theoretical considerations related to the thickness of the liquid solvent film adhering to the substrate as a function of its velocity [60–61]. Thus, besides the added block lengths of the di*block*-co-polymer forming the micelles, the dip-coating velocity adds yet another possibility to fine-tune the final inter-micelle distance of substrate supported carriers. In the case of CoCl_2_-loaded PS(1779)-*b*-P2VP(857) micelles, the interparticle distance can be tuned to between 80 nm and 140 nm.

In the previous section we derived the relationship between the final particle diameter and the interparticle distance 6*d* ≤ *D* from the simplest considerations. The closest interparticle distance observed in Figure 1 is about *D* = 70 nm while the final particle after etching (discussed below) is found to be *d* = 8 nm. Thus, the ratio *D*/*d* = 8.75 confirms our estimate. Similar experiments with shorter polymers (CoCl_2_-loaded PS(312)-*b*-P2VP(71) micelles, not shown) revealed a smallest interparticle distance of *D* = 18 nm at a final particle diameter of about 3 nm, in line with the given estimate of the correlation of interparticle distance and final particle diameter.

**Figure 2:**
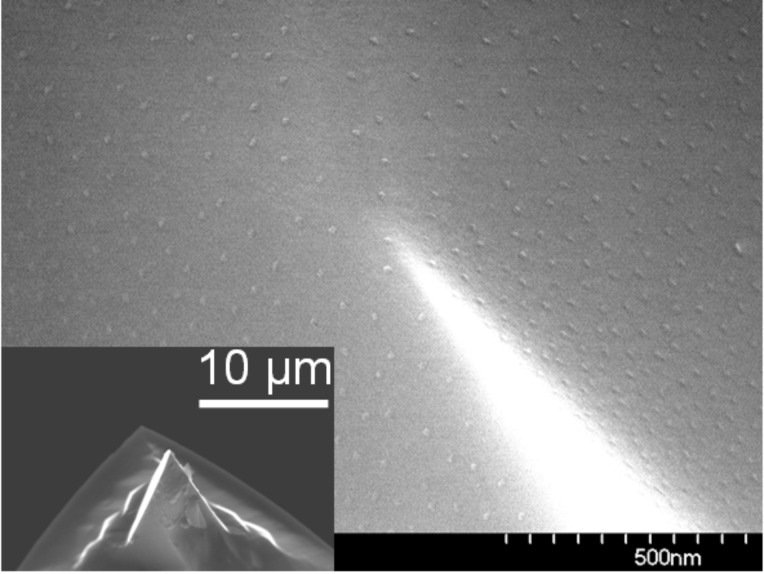
Co NPs prepared from CoCl_2_-loaded PS(1779)-*b*-P2VP(857) reverse micelles deposited on a Si AFM-tip by dip coating. The SEM image shows the base of the pyramid where Co particles homogeneously cover the root side (top part) as well as the side surfaces of the pyramid (lower part). The inset shows the AFM tip on the cantilever.

The deposition of arrays of micelles and subsequent NP fabrication is by no means restricted to planar substrates. This is demonstrated in Figure 2 where the SEM image shows Co NPs (average diameter 8 nm), prepared on the triangular pyramid surfaces of a Si AFM-tip (an overview of the tip shape is shown in the inset at the lower left of the figure). Even in this case, laterally quite homogeneous particle arrays could be realized. Note that the most homogeneous coverage is obtained when the AFM tip points upwards during the dip-coating process. In order to enhance the material contrast in SEM investigations, oxygen plasma is used for particle nucleation and for the removal of the polymers. This procedure is discussed below in greater detail.

#### 1.4 Plasma-assisted particle nucleation and reduction

Once the micellar carriers have self-organized into hexagonally ordered arrays during the dip-coating process on a substrate like those presented in Figure 1 (a, b), their role to provide an as high as possible degree of order is finished. The next decisive step is the complete removal of the organic carrier material and, simultaneously, to transfer the precursor into NPs without losing the previously established hexagonal order. The way to accomplish this involves exposure of the deposited loaded micelles to various plasma conditions [32,34]. For this purpose, a cluster of vacuum recipients consisting of a plasma (base pressure 10^–8^ mbar), analysis (base pressure 10^–10^ mbar), film deposition (base pressure 10^–8^ mbar) and sample storage chamber (base pressure 10^–10^ mbar) was designed and installed, all interconnected by transfer systems. Especially, the in situ transfer from the plasma into the analysis chamber allows an immediate X-ray photoelectron spectroscopy (XPS) investigation of the prepared NPs giving information on their chemical composition as well as on the presence of residues of the micelle matrix or on possible contaminations. It is worth noting that the plasma chamber together with its transfer system can be hooked up to a high-field end station at beam line PM3 at BESSY II synchrotron facility (Berlin), Germany and the 350 keV ion accelerator at Ulm University allowing full in situ sample manipulation.

After positioning the micelle containing substrate via a load lock system within the plasma chamber, an oxygen rf-plasma is ignited (frequency 13.56 MHz, operating pressure 4·10^–2^ mbar, power 50 W resulting in a dc self-bias of –500 V with the sample holder grounded). Simultaneously, a sample heater is started bringing the substrate temperature up to 250 °C or 300 °C for small or large micelles, respectively. This heating proved advantageous for the nucleation and growth process of the desired NPs. After an exposure time of typically 30 min, the oxygen plasma and heat treatment were stopped and the plasma chamber pumped down to its base pressure. Since the oxygen plasma treatment leads to oxidized NPs, for all magnetic NPs discussed in the present work an additional hydrogen plasma step was applied to reduce the particles into the metallic state. For this purpose, a hydrogen working pressure of 0.1 mbar was established in the plasma chamber and the plasma ignited. Again supported by heating the substrate up to 250 °C, the NPs were exposed to the hydrogen plasma for typically 20 min. Immediate transfer and XPS analysis revealed completely reduced metallic NPs. In the case of subsequent ex situ magnetic measurements on the NPs, they were coated in situ by thermal evaporation of SiO until a 10–20 nm thick layer had formed giving excellent protection against re-oxidation. It should be noted, however, that the NPs might as well be brought to ambient conditions to allow, e. g., their SEM characterization. In that case, the analysis is, of course, performed on oxidized particles, which has to be taken into consideration when determining their size. The particles, however, can be completely reduced to their metallic state by submitting them to the above described hydrogen plasma process. Once this state is established, all subsequent measurements have to be performed in situ or, alternatively, a protection layer has to be provided before exposing the NPs to ambient conditions.

#### 1.5 Preparation summary: Achievements and Limitations

**Figure 3:**
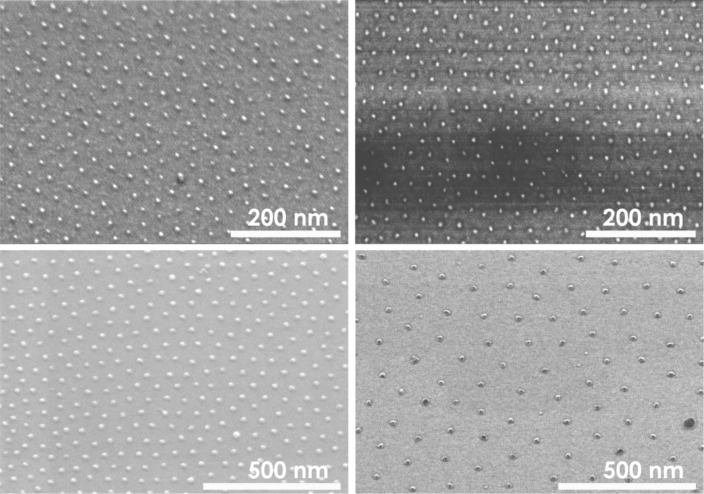
Fe (left column) and Co (right column) NPs prepared from PS(312)-P2VP(71) (upper panels) and PS(1779)-P2VP(857) (lower panels) reverse micelles. Note different scale bars.

In the preceding subsections details are given on how the “carrier principle” can be realized to obtain finally ordered arrays of metallic NPs. Although this preparation route is quite general, in the present contribution magnetic NPs are exclusively the focus. Accordingly, in Figure 3 examples are presented for arrays of elemental Fe and Co NPs demonstrating the high control of the procedure over particle size, interparticle distance and hexagonal order: The left panels show Fe NPs and the right panels Co NPs. The particles shown in the upper panels have been prepared from PS(312)-P2VP(71) resulting in about 3 nm particles whilst for the ca. 8 nm particles in the lower panels PS(1779)-P2VP(857), reverse micelles were used. Note, that the SEM images in Figure 3 show NP arrays obtained directly after the oxygen plasma treatment; consequently fully oxidized particles are shown. The corresponding interparticle distances of the NPs differ between the small and large NPs as can be seen from the different scale bars for the upper and lower panels, respectively. The sizes and interparticle distances found are within the general range accessible by the micellar method:

2 nm ≤ particle diameters *d* ≤ 12 nm

20 nm ≤ interparticle distances *D* ≤ 140 nm

**Figure 4:**
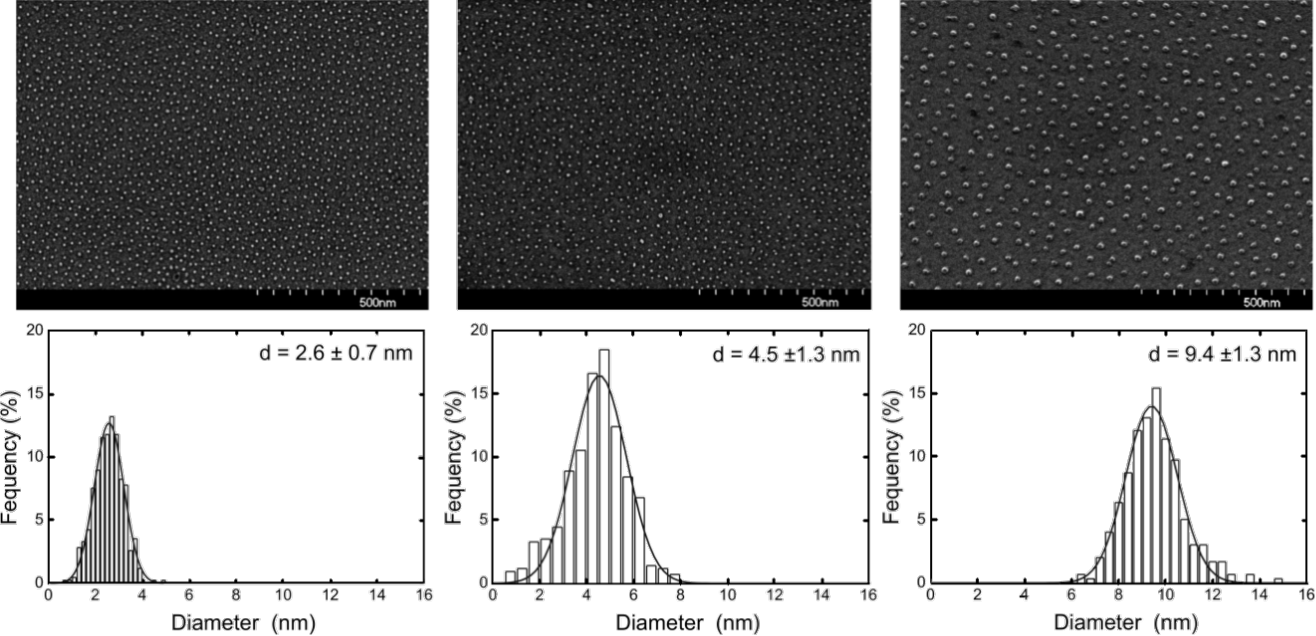
SEM micrographs of FePt NPs with 2.6 nm, 4.5 nm, and 9.4 nm diameter (top panels) and their AFM height distributions (lower panels). Details of the polymers employed and the interparticle spacing can be found in Table 1.

These parameter ranges hold for alloy particles such as FePt or CoPt and they represent natural limitations of the approach mainly related to the limited number of pyridine moieties within the core of single micelles [62] as well as to a maximum length of the PS-blocks without losing their spherical shape. SEM images in Figure 4 clearly reveal a short range hexagonal order of the nanoparticles for FePt NPs as previously proven [34]. The NP arrays, however, do not exhibit long range hexagonal ordering. The lower panels in Figure 4 show the corresponding AFM height distributions of the NP ensembles. NP height and its distribution were calculated from Gaussian peak fitting of AFM histograms. It has been shown previously that the shape of the particles can be assumed to be spherical [63]. FePt NPs were prepared with diameters in the range 2.5–10.5 nm and narrow size distributions as summarized in Table 1. Besides the polymer chain length, periodicity of particles, the NP diameter, the average number of atoms per NP and the average NP composition (measured by XPS) are shown. The number of atoms per particle was calculated from the average diameter and bulk FePt lattice constant assuming perfect spheres. The integral composition of all NP batches as determined by XPS show Fe and Pt content close to the equiatomic composition and well within a range in which L1_0_ order of FePt is favored in the bulk (40–55% of Fe). From Table 1 it is striking that identical polymers, i.e., PS(1779)-*b*-P2VP(695) may form different-sized hydrophilic cores and consequently, the resulting particle sizes differ from one another. The effect is ascribed to the preparation under ambient conditions. Generally, we find larger final particle diameters during summer when air humidity is higher when some water may enter the toluene based micelle solution.

**Table 1:** Number of monomer PS-*b*-P2VP units, periodicity observed on Si/SiO_2_ after dip coating at an emerging velocity 15 mm/min, diameter of FePt NPs after nucleation, resulting number of atoms per NP and integral composition of the samples as determined by XPS.

Polymer PS[x]-P2VP[y]	Periodicity [nm]	Diameter [nm]	No. of atoms	Composition

266-41	30 ± 5	2.6 ± 0.7	570	Fe_48_Pt_52_
312-74	26 ± 5	2.7 ± 0.9	720	Fe_47_Pt_53_
266-41	28 ± 6	4.5 ± 1.3	3300	Fe_44_Pt_56_
1779-695	48 ± 11	5.8 ± 2.6	7200	Fe_49_Pt_51_
528-177	43 ± 10	6.3 ± 1.4	9200	Fe_50_Pt_50_
1779-695	55 ± 12	6.6 ± 1.7	10500	Fe_48_Pt_52_
1779-695	61 ± 12	9.4 ± 1.3	30400	Fe_44_Pt_56_
1779-857	68 ± 14	10.5 ± 2.5	42400	Fe_56_Pt_44_

Though the nanoparticle preparation via salt-loaded reverse micelles has been successfully performed on various types of substrates – dielectric and metallic, single crystalline and amorphous – some further restrictions related to their materials should be mentioned. First of all, to obtain NPs exposure to oxygen and/or hydrogen plasmas is necessary and the substrates must be able to withstand this etching procedure. In this context, among dielectric materials especially, oxides such as MgO, sapphire, SrTiO_3_, quartz were found to be suitable, as well as materials forming thin oxide layers such as Si. Furthermore, adhesion of the NPs is an issue. For the magnetic metal particles studied here, the following hierarchy of adhesion strengths was observed: sapphire < MgO < SiO_x_ (native Si-oxide). To arrive at this sequence, the force necessary to move the particles by the tip of an AFM was determined. In all cases, however, the supported particles could be annealed up to 700 °C without losing their positional order, i.e., no Ostwald ripening was observed.

A last remark addresses the effect of the hydrogen plasma treatment on NPs with a propensity for hydride formation such as in case of Co. Though the consequences of CoH_x_ formation on the corresponding magnetic properties are by no means clear-cut [64], one nevertheless has to take this into consideration. Indeed, close inspection of in situ X-ray absorption spectra (XAS) on Co NPs immediately after reduction in a hydrogen plasma revealed a significant shoulder 2 eV above the Co-L_3,2_ absorption maxima caused by hydrogen uptake. In this particular case, however, the hydrogen could be expelled by annealing at 650 °C for 5 min [65], and led to complete disappearance of the above XAS shoulder. However, there is no general rule on this issue and hydrogen uptake during the reduction step must be checked individually for each type of NP.

#### 2 Structural and chemical analyses

When the magnetic properties of NPs are investigated, the question immediately arises if and how the observation differs from the bulk properties and, moreover, how the observations can be correlated to (i) the structure of the particles and (ii) to the chemical state. Advanced analytical tools such as aberration-corrected high-resolution transmission electron microscopy (HRTEM) and related techniques as well as X-ray photoelectron spectroscopy (XPS) give important additional information on the atomic and electronic structure of the sample. Moreover, due to its surface sensitivity, XPS can be used to obtain information on surface oxidation, phase separation and segregation both in films and in particles [66]. In this section some important findings are discussed which have impact on the interpretation of the magnetic characterization.

#### 2.1 Characterization of the particle nucleation

**Figure 5:**
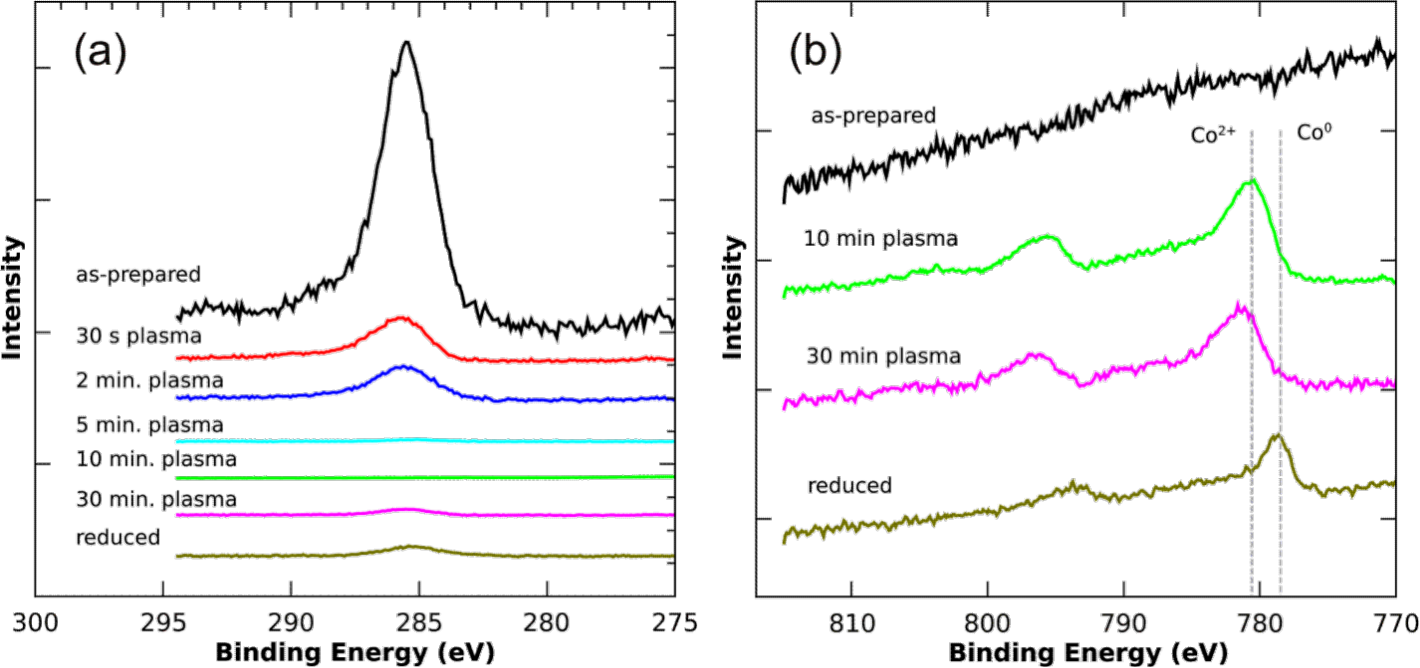
Photoelectron spectroscopy of Co-precursor loaded PS(1779)-P2VP(857) reverse micelles after different O and H plasma treatments. Details are given in the text; (a) and (b) show C-1s and Co-2p levels, respectively. All spectra are normalized to the Si-2p substrate signal intensity (not shown) and vertically off-set relative to each other for clarity.

In order to gain some insight of the particle nucleation and reduction processes by means of plasma treatment, as discussed in section 1.4, a series of C-1s and Co-2p XP spectra after different etching steps were measured for CoCl_2_ loaded PS(1779)-P2VP(857) reverse micelles. All spectra shown in Figure 5 are normalized to the total Si-2p signal intensity of the substrate. In the initial state PS-P2VP molecules dominate the survey scan proving the continuous coverage of the Si substrate by reverse micelles. The Co precursor material located in the cores of the micelles is not detected as a result of the surface sensitivity of XPS. Additionally, the development of the C and Co signals after different etching steps is shown in Figure 5. It is obvious that the C-1s intensity, which is predominantly related to the micelle shell, strongly decreases relative to the Si substrate even after oxygen exposure for only 30 s. After 5 min exposure time, the C-1s intensity dropped below the detection limit, while after 10 min a clear Co^2+^ spectrum can be observed. At this stage the particles simultaneously nucleate to form Co oxide NPs. To guarantee removal of the (small) volume between each nanoparticle and the substrate, which principally remains undetected by XPS, the etching time is typically tripled at temperatures up to 250 °C. After this etching period, the SEM images presented above were taken. In a final step, subsequent hydrogen plasma treatment (20 min) allows the reduction of the NPs into the metallic state as indicated by the Co-2p peak shift towards the position of metallic Co. Finally, it is worth noting that the small C-1s signal visible in the reduced state is due to the necessarily long acquisition time for the XPS spectra. For example, to arrive at a reasonable signal-to-noise ratio for the Co-2p peaks, a data acquisition time of 12 h is required. Even under UHV conditions such a long exposure of a sample surface to X-rays results in the built-up of a small amount of carbon contamination, which, by covering the Co particles, may result in a slightly reduced Co-2p signal.

#### 2.2 Oxidation of FePt nanoparticles

Degradation of magnetic properties due to oxidation is an important issue especially for applications. For naked 3d elemental nanomagnets (Fe, Co, Ni) as well as for alloys (e.g. FePt or CoPt) one can expect a strong deterioration of the targeted magnetic properties under ambient conditions [67–68]. For FePt alloy NPs, we recently investigated the oxidation behavior in more detail by XPS and signal modeling taking into account the spherical shape of NPs [66]. Here, we briefly summarize the results.

**Figure 6:**
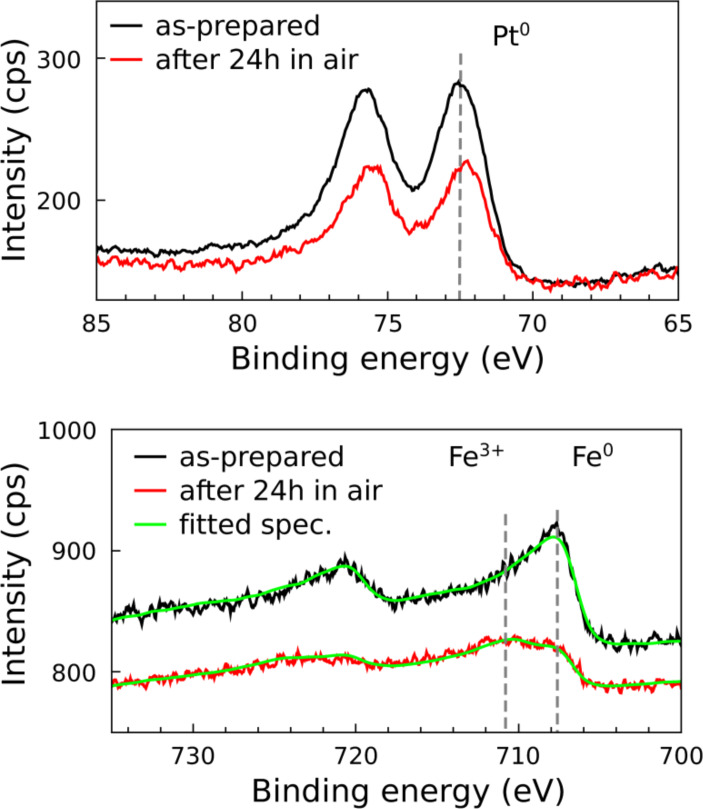
Pt-4f and Fe-2p XPS spectra of 9.8 nm FePt particles before and after exposure to ambient air for 24 h (Fe-2p spectrum is vertically off-set for clarity). After exposure the majority of Fe atoms are found in the Fe^3+^ state, while Pt atoms remain metallic. All spectra were smoothed by 3-point moving window averaging and no further normalization was performed. Green solid curves through Fe-2p spectra in the lower panel are fits based on Fe^0^ and Fe^3+^ reference spectra.

Figure 6 shows Pt-4f and Fe-2p XP spectra of (9.8 ± 0.6) nm FePt NPs in the metallic state and after 24 h exposure to air. Oxidation in ambient air becomes obvious by a clear energy shift of the Fe 2p_3/2_ core level to about 711 eV with a shoulder at the metal position still present. By comparison with literature data the main peak can be assigned to the Fe^3+^ oxidation state [69]. Interestingly, Pt-4f levels reveal no significant indication of oxide formation, proving the chemical state of the Pt atoms remains practically unchanged during oxidation of the FePt particles. This finding is astonishing, since it is well known that a thin Pt oxide layer forms on pure Pt under ambient conditions [70–71]. Although Pt-4f spectra have not been normalized before and after the oxidation process, e. g., to the total Fe-2p intensity, there appears to be a reduction of Pt-4f intensity which might easily be explained by the formation of a thin Fe oxide overlayer damping the Pt intensity. More details can be found in [66], in which we quantified the degree of oxidation by XPS line fitting using linear combinations of reference spectra measured under identical experimental conditions. The Fe^0^ spectrum was obtained from a reduced and, thus, completely metallic FePt thin film sample, while the Fe^3+^ spectrum is measured on pure (99.5%) bulk Fe after complete oxidation in oxygen plasma. The outcome of such fitting is also shown in Figure 6 by the green solid lines.

More detailed investigations on the relative amount of Fe oxide formed after exposure to molecular oxygen and air for differently sized nanoparticles in the as-prepared metallic state have shown that a minimum oxygen dose of 10^6^–10^7^ L is necessary to observe a Fe^3+^ signal above the detection limit. Higher gas exposures result in a logarithmic increase of the ratio of Fe^3+^ to Fe^0^ intensities [66].

After annealing at 650 °C for 90 min of 9.8 nm and 11.5 nm NPs, thus partially L1_0_ ordered, the oxidation rate drastically decreased. More quantitatively, annealed FePt NPs withstand 100–1000 times longer exposures to molecular oxygen than their non-annealed counterparts. A completely different oxidation behavior was displayed by 4.9 nm FePt NPs for which only low oxygen doses were needed to obtain the oxidized state and the oxidation rates were practically identical for as-prepared or annealed NPs [66]. Both types of oxidation behavior as exhibited by larger or smaller FePt NPs can be consistently described by Pt segregation towards the particle surface.

#### 2.3 Pt segregation in FePt nanoparticles

For icosahedral FePt NPs, recent HRTEM observations have indicated a systematic increase of the lattice parameter towards the periphery of the particles starting with the bulk value in their interior [72–73]. This finding strongly points to Pt segregation towards the particle surface. A more direct way of testing for such Pt segregation is the application of an element-specific surface sensitive technique such as XPS.

Based on a FePt core–Pt shell model, the observed Pt-4f and Fe-2p spectral intensities can be analyzed quantitatively to confirm Pt segregation and give for 4.9 nm FePt NPs a value of the Pt surface enrichment of less than an equivalent of 0.1 nm pure Pt. Experimentally, we observed that after annealing 9.8 nm FePt NPs at 650 °C for 90 min the intensity ratio I(Fe)/I(Pt) droped to almost half the value observed for the as-prepared sample [66]. From this, Pt shell thicknesses of 0.2 nm and 0.3 nm were obtained for 11.5 nm and 9.8 nm NPs, respectively, while for a FePt reference film the value was 0.27 nm. Thus, the larger particles and the film gave similar results. These findings immediately suggest that in case of the film and the larger NPs, a Pt surface layer approximately one monolayer thick was formed which, in turn, strongly impedes further oxidation. For 4.9 nm FePt NPs (and smaller) this Pt surface layer is no longer complete and thereby loses its protecting effect. This latter behavior may have its origin in the strong compositional change within the interior of the particle induced by Pt segregation. As an example, for 4.9 nm NPs, a complete Pt shell with thickness of one monolayer segregated from the interior would shift the atomic composition of the core from Fe_53_Pt_47_ to Fe_68_Pt_32_. According to the FePt bulk phase diagram, such a compositional change would lead to a structural transformation into the Fe_3_Pt phase. In this case, the driving force of a decreasing total surface energy due to Pt segregation is probably overcompensated by the energy needed to form Fe_3_Pt from FePt.

#### 2.4 Structure of FePt nanoparticles

Since the magnetic hardness of FePt alloys strongly depends on the chemical order parameter, we investigated the structure of individual particles by aberration corrected HRTEM. While HRTEM and electron diffraction does not provide absolute quantification of the ordering parameter as can be achieved by scanning (S)TEM at atomic resolution at its mass sensitive contrast [74], it allows a relatively fast way to distinguish between ordered and disordered phases. For the purpose of HRTEM investigations, 3 nm and 8 nm FePt NPs were prepared on Si/SiO_2_ substrates. (Partial) chemical order was established by annealing at typically 650 °C for 90 min. Prior to TEM investigations, selected samples were covered with a protective layer of about 10 nm SiO_2_ to avoid oxidation and mechanical damage due to TEM lamella preparation. The samples were cut into pieces (diamond wire saw), mechanically ground, dimpled, and polished to a thickness of <5 µm (Gatan dimple grinder). Low angle (10°) argon ion etching with energies of 5 to 1 keV (Fischione 1010 ion mill) was used to achieve electron transparency with lamella thicknesses smaller than 100 nm. TEM investigations were carried out using a FEI Titan 80-300 microscope operating at 300 kV equipped with a CEOS type imaging aberration corrector and a slow scan CCD camera system. The aberrations were corrected up to the 3^rd^ order resulting in a phase plate of 20 mrad (π/4 criterion) and a point to point resolution down to 0.1 nm.

**Figure 7:**
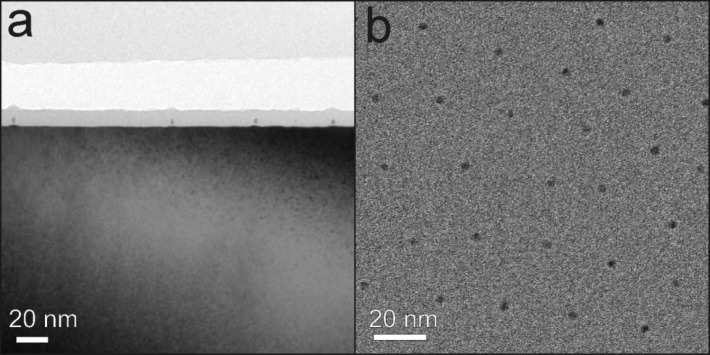
TEM images of 3 nm FePt NPs on Si/SiO_2_ after annealing at 650 °C for 90 min. (a) Bright-field TEM image of FePt nanoparticles in cross section view. The NPs are in situ covered by a thin layer (10 nm) of SiO_2_ to avoid oxidation and mechanical damage after annealing. (b) Bright-field TEM image of FePt NPs in plane view. The hexagonal ordering can be clearly seen.

Figure 7 shows overview TEM images of 3 nm FePt on Si/SiO_2_ in cross section (a) and plane view geometry (b) after annealing at 650 °C for 90 min. The hexagonal 2D ordering of the particles is clearly visible in Figure 7 (b) and compares well with the SEM imaging in Figure 4.

**Figure 8:**
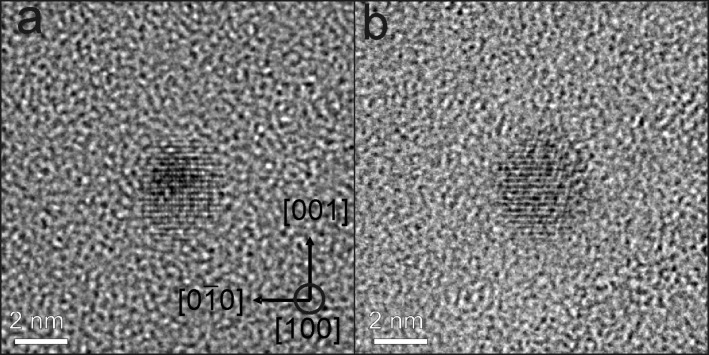
Aberration corrected HRTEM images of FePt particles seen along [100] direction. The L1_0_ ordering along [001] axis can be identified by the super lattice planes.

The structure as well as the crystallographic orientation relative to the beam direction of NPs was determined by aberration corrected HRTEM when the remaining substrate/embedding film thickness made them accessible. L1_0_ ordering of 3 nm FePt NPs is demonstrated in Figure 8. Here, two examples are presented in which the NPs are oriented along [100] direction. The presence of superlattice planes along the [001] axis proves the presence of L1_0_ phase. Moreover, the particle in Figure 8(a) appears nearly spherical exhibiting no obvious defects, twins or stacking faults while the NP in Figure 8(b) shows L1_0_ order at the left as well as a strongly distorted region (right).

**Figure 9:**
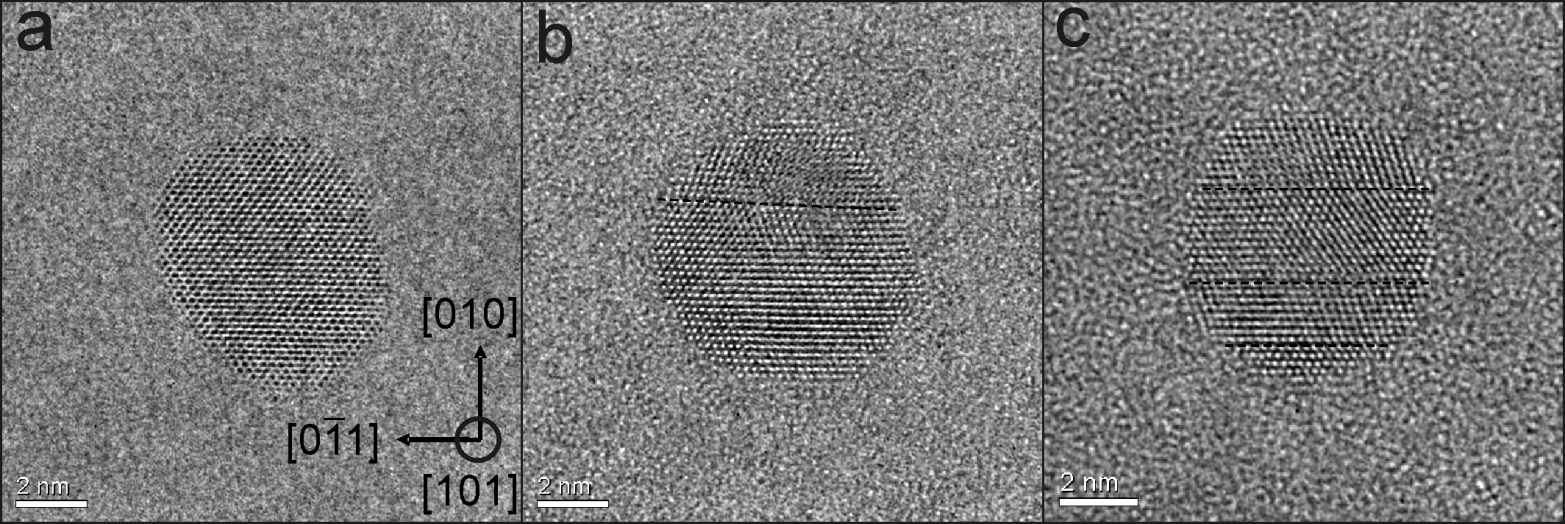
Aberration corrected HR-TEM images of about 8 nm FePt NPs in [101] orientation. Crystal defects such as twins or stacking faults can clearly be seen along [101] projection (defects are marked by the broken line in (b,c)). Only few particles such as in (a) do not exhibit stacking faults. Most particles contain one (b) or more (c) stacking faults.

The presence of possible crystal defects such as twin and stacking faults reducing the degree of chemical order and, as a consequence, leading to a reduced magnetic anisotropy, were easiest to see along the [101] direction. Figure 9 demonstrates that especially larger particles (8 nm) can exhibit twins (Figure 9(b)) and stacking faults (Figure 9(c)). Particles without defects, however, were also present (Figure 9(a)). The statistical analysis of in total 70 NPs (3 nm) shows that 38 FePt NPs exhibit defects along the [101] direction. From this result and the fact that additional defects may exist which cannot be seen in the [101] projection, it can be concluded that for the majority of particles crystal defects are a common feature. For 8 nm FePt NPs defect-free NPs are rare. In turn, this finding already suggests that the effective magnetic anisotropy energy density may be lowered compared to bulk FePt single crystals. In section 3.3.2 this issue will be addressed.

### 3 Magnetic properties

#### 3.1 General remarks on the magnetic characterization

The principal difficulty in investigating the magnetic properties of NPs prepared by reverse micelles poses is having to deal with small magnetic signals composed of contributions from both, the NPs as well as the supporting mostly diamagnetic substrate. For example, 8 nm Co NPs with a bulk saturation magnetization of 1.73 µ_B_ per atom and interparticle distances of 100 nm on a 5 **×** 5 mm^2^ substrate produce a total magnetic moment of only 10**^–^**^9^ Am^2^ (10**^–^**^6^ emu). Although state-of-the-art SQUID-magnetometry is able to detect the related small signals, the response of the diamagnetic substrate has to be taken into account as well delivering for the typically applied external fields signals of the same order of magnitude as the particles, however, of opposite sign. Thus, the magnetic responses from the NPs and the substrate cancel each other at external fields between 20**–**200 mT depending on the volume of the substrate and diamagnetic susceptibility. Consequently, SQUID-magnetometry on supported micelle-based NPs is strongly hampered and often not reproducible. Moreover, prior to an ex situ characterization, the as-prepared particles have to be in situ coated, e.g., by SiO_2_ to avoid oxidation under ambient conditions. Even then, further sample processing such as the annealing of the covered NPs appears unfavorable due to the possible occurrence of chemical reactions. If, however, all these problems are carefully considered, SQUID-magnetometry can provide valuable information.

**Figure 10:**
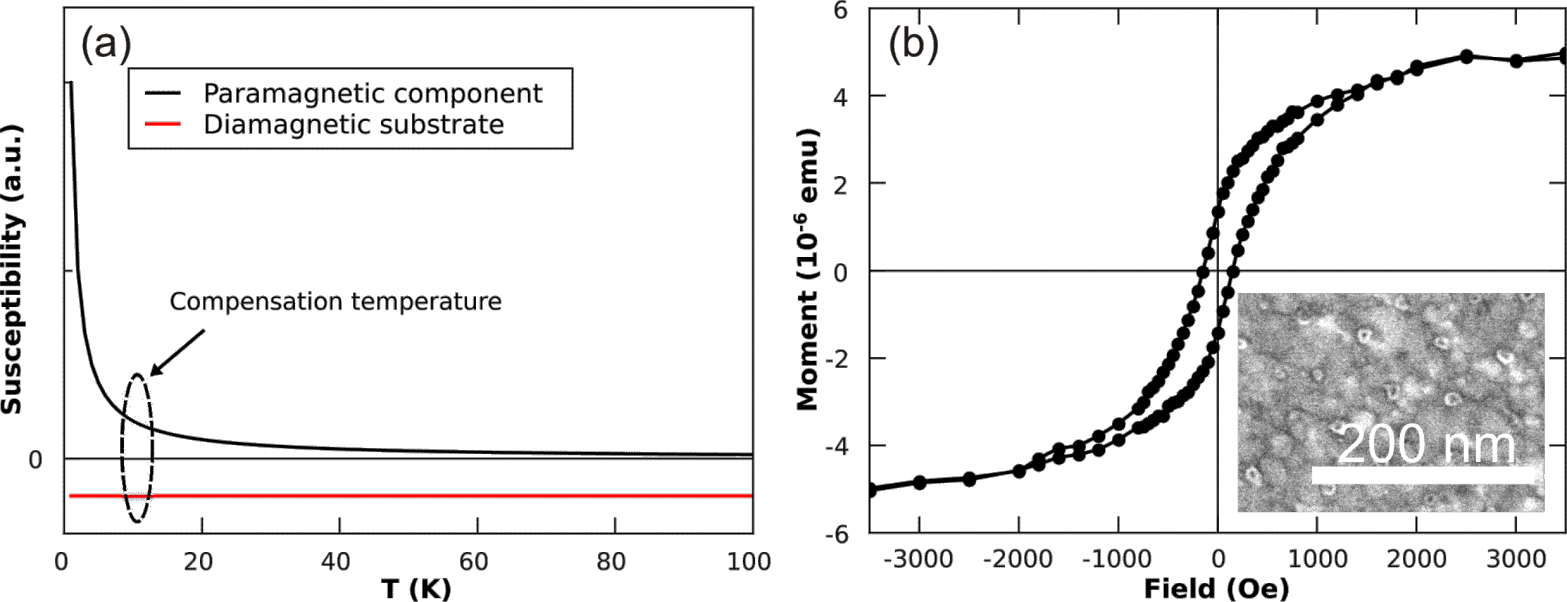
(a) Schematics of the compensation approach: Temperature dependence of the magnetic susceptibility of diamagnetic substrate and paramagnetic thin film deposited on the sample. At a certain temperature, which can be adjusted by the film thickness of a paramagnetic film, the two components compensate each other. (b) In-plane hysteresis loop of 8 nm Co particles deposited on a polycrystalline 50 nm Pt(111) film on MgO(001). The compensation temperature of film and substrate is found at T = 29 K. The inset shows a SEM image of Co particles prepared on the Pt(111) film.

The drawback of a strong diamagnetic substrate contribution in SQUID-magnetometry can be circumvented to some extent by use of a paramagnetic film to compensate for the temperature-independent diamagnetic contribution. In Figure 10(a) this approach is demonstrated. Once the diamagnetic response of the substrate has been determined or evaluated from susceptibility data, the necessary thickness of a paramagnetic film can be easily calculated. Note that the calculation only gives an estimate and the actual sample compensation temperature has to be determined separately for each sample due to slight variations in the substrates, film thicknesses and possible impurities.

#### 3.2 Co nanoparticles on Pt films

The approach of compensating diamagnetic substrate signals by paramagnetic films is demonstrated by paramagnetic Pt(111) films on top of MgO(001) substrates. Pt films were deposited under UHV conditions at ambient temperature by pulsed laser deposition (PLD). For this purpose a 193 nm ArF laser was employed hitting a Pt target (purity 99.99%) at typical areal energy densities of 8 J/cm^2^. Details of the setup are described elsewhere [63,75]. The 50 nm polycrystalline Pt films exhibited a (111)-texture and a mean grain size of about 10 nm. Subsequent micelle deposition and plasma etching (details are given in section 1.4) led to Co NPs with an average particle height of 8 ± 1 nm (measured by AFM) with an interparticle distance *D* ≈ 60 nm (see inset of Figure 10 (b)). The measured hysteresis loop showed a sample saturation moment, remanent magnetization and a coercive field of *M*_S_ = 5∙10**^–^**^6^ emu, *M*_R_ = 26%, H_C_ = 150 Oe, respectively. This value of the coercive field is typical for Co NPs [32]. An *M*_R_ of 26%, which is only about half the value expected for Stoner-Wohlfarth NPs, suggests that already a significant amount of NPs is in the superparamagnetic state at *T* = 29 K. Comparing the saturation moment with the considerations mentioned above, we expected a total sample magnetic moment of 2.8∙10**^–^**^9^ Am^2^ (2.8∙10**^–^**^6^ emu) taking into account the NPs density at an average distance of 60 nm. Although this estimate is 44% lower than the experimental value, this deviation is acceptable taking the error bar of the volume of NPs into account.

**Figure 11:**
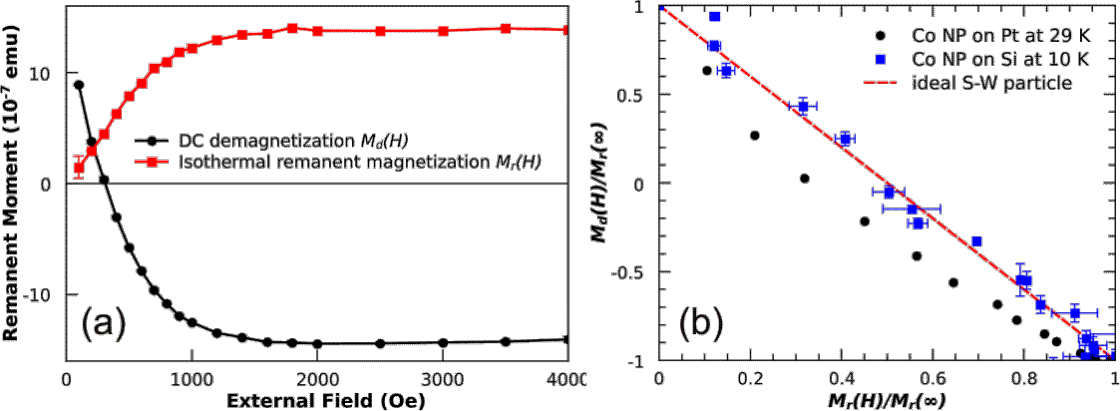
(a) DC-demagnetization and isothermal remanent magnetization of 8 nm Co particles measured by SQUID-magnetometry at *T* = 29 K in in-plane geometry; (b) shows the so-called Henkel plot of the Co NPs on Pt(111) film on Si (black dots) and the result obtained for Co NPs on a Si substrate at *T* = 10 K (blue squares). The expectation for ideal Stoner**–**Wohlfarth particles (red line) at *T* = 0 K is also included.

Alternatively, NP ensembles exhibiting a remanent magnetization, i.e., the blocked state, can be characterized in zero external fields leading to vanishing substrate and film signals. The combination of DC-demagnetization (DCD) and isothermal remanent magnetization (IRM) [76–77] can give additional information of possible magnetic interaction of the NPs. Figure 11 (a) presents such a measurement on 8 nm Co particles at *T* = 29 K. The external field was applied in the substrate plane. The remanent magnetization is plotted as function of external field applied before measuring in zero fields the DCD and IRM dependencies. By combining the two measurements, a corresponding Henkel plot can be obtained. For ideal Stoner**–**Wohlfarth NPs the Henkel plot would yield a straight line with slope of –2. The result of 8 nm Co NPs on the Pt film was found to be below this Stoner–Wohlfarth limit. This deviation can be understood within the framework of thermal excitation as previously shown [76]. From a qualitative standpoint, the thermal energy gives rise to a reduced switching field of NPs and thus a steeper slope of the DCD and a lowered increase in the IRM curves, respectively. As a consequence, the Henkel plot for non-interacting particles measured at finite temperatures runs below the one expected for ideal Stoner**–**Wohlfarth NPs (straight line in Figure 11 (b)). For comparison, the Henkel plot of 8 nm Co NPs on Si substrates is also included in Figure 11 (b) which nicely demonstrates the validity of the approach. Note that the larger error bars of Co NPs on Si substrate are due to the lower saturation magnetization of the sample (10**^–^**^9^ Am^2^).

#### 3.3 FePt alloy particles

For the magnetic characterization of FePt alloy particles we chose X-ray magnetic circular dichroism (XMCD) which provides information on (i) the chemical state of sample, (ii) element-specific magnetic moments and (iii) element-specific hysteresis loops. The results presented below were measured at beamline PM-3 of the BESSY II synchrotron facility in Berlin, Germany. The total electron yield was recorded as function of photon energy in external fields up to 3 T and at variable temperatures between 11 K and 300 K [78]. Due to its surface sensitivity, it becomes possible to measure X-ray absorption spectra and hysteresis loops with high precision, even of NPs. Note that the XMCD and hysteresis loops were always measured in out-of-plane geometry. Moreover, our home-built plasma etching system can be attached to the high-field end-station which allows full in situ sample manipulation and characterization [32].

#### 3.3.1 Tracking the phase transition in FePt nanoparticles

**Figure 12:**
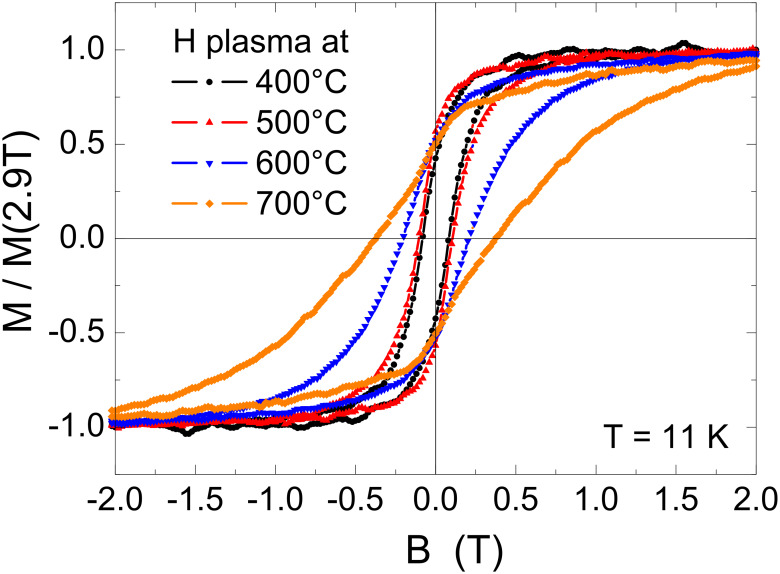
Element-specific magnetic hysteresis loops of 5.8 nm FePt NPs taken at the Fe-L_3_ edge at *T* = 11 K after different annealing steps for 30 min annealing time in hydrogen plasma. Note that hysteresis loops are normalized to the magnetization at *B* = 2.9 T (maximum field).

The setup described above enabled us to study the impact of chemical ordering on the magnetism of FePt alloy particles. For this purpose, element-specific hysteresis loops of FePt particles with an average diameter of 5.8 nm were recorded at T = 11 K after different annealing steps in hydrogen plasma. The hysteresis was extracted from the total electron yield signal as previously described [78]. It is important to note that the size distribution does not change due to annealing (for details see e.g. [79]) and thus changes of magnetic hysteresis can be attributed to changes of the effective magnetic anisotropy energy density *K*_eff_ originating from variations of the chemical order within the particles. Figure 12 shows the experimental results for successive annealing steps on the same sample from 400 °C to 700 °C in 100 K steps. It is obvious that the initial annealing steps at 400 °C and 500 °C do not significantly change the magnetism of the FePt particles. The coercive field *µ*_0_*H*_C_ is around 0.1 T while the particles remain superparamagnetic at *T* = 300 K (not shown). After annealing at 600 °C (700 °C), *µ*_0_*H*_C_ gradually increases to 0.2 T (0.38 T) proving the increasing chemical order. Interestingly, the remanent magnetization *M*_R_ is found at (50 ± 5)% of the saturation magnetization for all annealing steps. This value is expected for non-interacting Stoner–Wohlfarth particles with randomly oriented, uniaxial anisotropy axes [1]. In section 3.3.2 we make use of this model to deduce *K*_eff_ from hysteresis loops.

**Figure 13:**
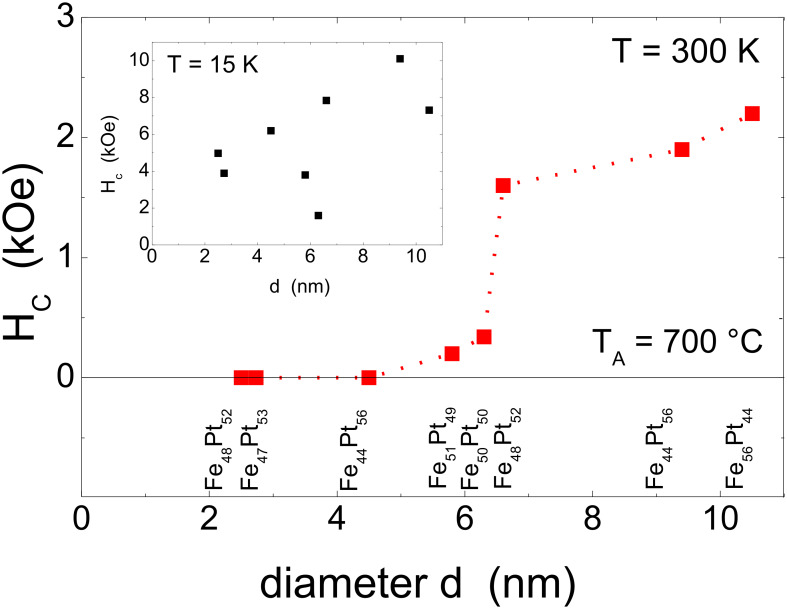
Coercive field at *T* = 300 K as function of the diameter of FePt NPs after annealing for 30 min at *T* = 700 °C. The dotted line serves as a guide to the eye. The particle compositions as determined by XPS are noted for each array of particles. The inset shows the coercive fields at *T* = 15 K.

Changes of the magnetic hysteresis of FePt NPs were also investigated as function of particle diameter. For this purpose NPs were prepared on Si/SiO_2_ substrates by reverse micelles. See Figure 4 and Table 1 for details. Figure 13 shows the experimental coercive field as function of FePt NP diameter at *T* = 15 K (inset) and *T* = 300 K after annealing at *T*_A_ = 700 °C for 30 min. At low temperature, the coercive field *H*_C_ was found between 1.6 kOe and 10.1 kOe thus scattering to high extent. Leaving the experimental points at 5.8 nm and 6.3 nm for a moment, a continuous increase of *H*_c_ as function of NP diameter was found. The excluded 5.8 nm and 6.3 nm FePt NPs exhibit a significantly different shape of their hysteresis curves with a notably narrow waist corresponding to significantly reduced *H*_c_ values. This feature will be discussed in the next section in more detail. More importantly, at 300 K the coercive field follows a similar dependence on the NP diameter. Small NPs are superparamagnetic and only for diameters larger than 6 nm a non-zero coercive field was observed at *T* = 300 K. For 6.6 nm, 9.2 nm, and 10.5 nm FePt NPs large coercive fields of 1.6 kOe, 1.9 kOe, and 2.2 kOe at ambient temperature, respectively, were found.

These results directly imply that the magnetic blocking of NPs is size-dependent at 300 K. For applications, a standard requirement for the orientation stability of blocked particle magnetizations is given by 30 k_B_T ≤ *K*_eff_ ∙*V* assuming the time window of the XMCD-based hysteresis measurement (600 s). With the results from Figure 13 which indicate that only FePt NPs above ca. 7 nm show strong hysteretic behavior, it is possible to calculate the minimum *K*_eff_ yielding blocked NPs. In this way, a value of *K*_eff_ = 0.69 MJ/m^3^ at *T* = 300 K was found which is about one order of magnitude smaller than the bulk FePt anisotropy constant. Moreover, it is interesting that the observed *H*_c_ values strongly scatter around a NP diameter of 6 nm at *T* = 15 K. This finding, however, cannot be attributed to integral composition variation of FePt NPs as indicated by the XPS results in Table 1. Rather, the compositional variation of individual particles may cause the observed behavior. Changes in the shape of hysteresis loops are discussed in the next section in more detail.

#### 3.3.2 Magnetic anisotropy of FePt nanoparticles

The direct determination of magneto-crystalline anisotropy energy densities *K*_MC_ from experiments can be carried out by several techniques: ferromagnetic resonance (FMR), XMCD or alternatively, SQUID magnetometry using magnetic hysteresis loops or zero-field/field cooling (ZFC/FC) temperature scans. In all cases advanced models must be applied to fit the experimental data correctly. Generally, ferromagnetic resonance [80] appears as the simplest and most reliable technique in case the sample can be saturated during a full angular scan varying in-plane and out-of-plane angles Φ and θ, respectively. Note, that also the first and second *K*_MC_-constants should be rather small (just as for the elemental magnets Fe, Co, and Ni) to observe resonances close to the magnetic hard axis in accessible external fields of a standard electromagnet. If a magnetic film has been grown epitaxially with a well defined single crystalline structure, it becomes possible to determine anisotropy constants with high precision in different crystallographic directions [81]. XMCD relies on the precise determination of the anisotropy of orbital magnetic moments Δμ_L_ in the direction of easy and hard axes of magnetization. It has been shown both by theory [82] and experiment [83], that Δμ_L_ can be linked to *K*_MC_-constants. Both techniques, however, are more difficult to apply on systems with random distribution of anisotropy axes as in case of NPs discussed in this article [84]. However, some efforts have been undertaken to obtain information on an effective anisotropy constant *K*_eff_ in which all possible contributions such as surface effects [85] or composition effects [53] to anisotropy are combined in a single, effective constant [86].

It is well known that ZFC/FC measurements can be used for an estimate of *K*_eff_ [53] employing the total anisotropy energy by *E*_aniso_ = *K*_eff_ ∙*V*. In other words, for the volume of NPs a distribution is assumed while *K*_eff_ is taken as a constant. For monatomic NPs this procedure appears quite successful and could easily be extended to small particles where surface anisotropy plays an important role using *E*_aniso_ = *K*_V_ ∙*V* + *K*_S_ *S*, where *S* is the surface area [85].

In case of magnetic alloy NPs such as FePt or CoPt, however, compositional distributions as well as distributions of the degree of chemical order may significantly determine the magnetic properties. Thus, these parameters have to be considered for estimates of *K*_eff_. Practically, a *K*_eff_ distribution is included into fit formulas of ZFC/FC measurements as recently shown [53]. Alternatively, for non-interacting NPs with uniaxial anisotropy, the combination of low temperature experimental hysteresis loops and simulations along the Stoner–Wohlfarth model [1] introducing a *K*_eff_ distribution can be applied. As opposed to ZFC/FC measurements this approach does not include thermal energies to which *E*_aniso_ is compared. Moreover, *K*_eff_ and its distribution are the fitting parameters for simulations of magnetic hysteresis loops and volume distributions do not enter.

The above described approach is tested first on hysteresis loops of 5.8 nm NPs. Initially, a Gaussian shaped distribution of *K*_eff_ was assumed in-line with a recent report by others [53]. It turned out, however, that this approach delivered a satisfactory description of the hysteretic behavior only for as-prepared FePt NPs immediately after in situ reduction, i.e., for fcc NPs exhibiting a low anisotropy. In contrast, after annealing it is not possible to fit the experimental hysteresis loops by a single Gaussian for *K*_eff_. This finding is most naturally explained by the presence of two types of NPs: (i) a low anisotropy component accounting for particles which do not reach high *K*_eff_ values by annealing. The low *K*_eff_ for a (small) portion of NPs could be attributed to composition variations larger than the window in which L1_0_ order is favoured; (ii) the desired high anisotropy component that shifts to larger values by the annealing induced formation of the ordered L1_0_ phase. Consequently, a bimodal Gaussian-shaped distribution is applied for the simulation:

[1]
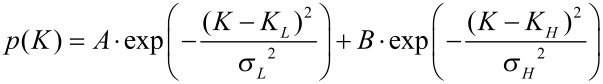


**Figure 14:**
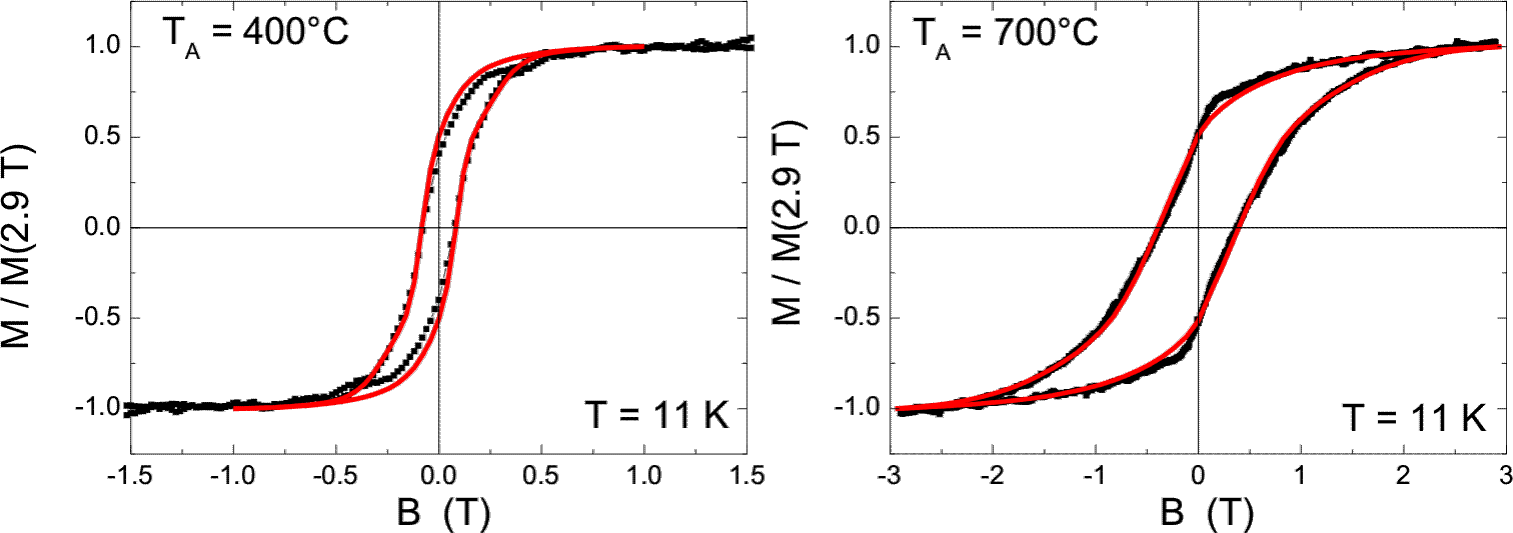
Experimental hysteresis loops of 5.8 nm FePt NPs at 11 K and simulations using a bimodal Gaussian *K*_eff_ distribution (as discussed in the text) after annealing at *T*_A_ = 400 °C and *T*_A_ = 700 °C, respectively.

Here *p*(*K*) is the probability to find a value *K* for anisotropy, *A* and *B* account for peak weights of the two Gaussians centered at *K*_L_ and *K*_H_ while σ_L,H_ denote the corresponding standard deviations. Simulations with varying parameters are applied until a reasonable congruence has reached. Figure 14 presents two examples of the results of such fitting for FePt NPs after annealing at *T*_A_ = 400 °C and 700 °C. Notably, the experimental data are nicely reproduced when using a bimodal Gaussian distribution.

**Figure 15:**
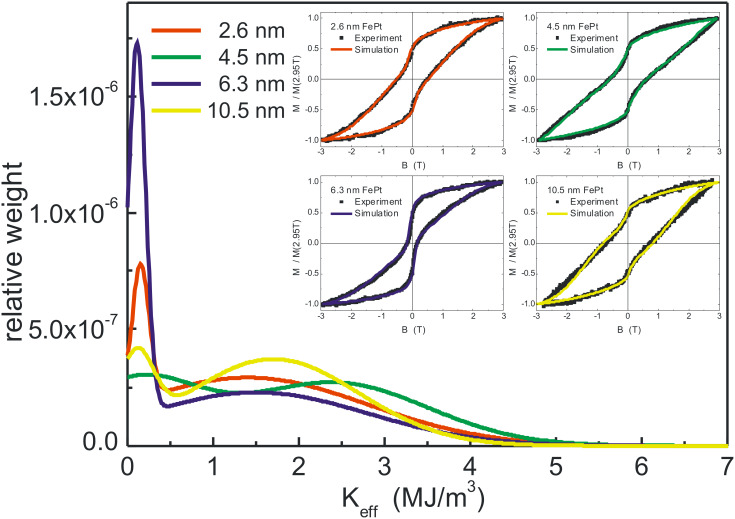
Distributions of the effective anisotropy *K*_eff_ for arrays of differently sized NPs used for simulating the experimental low temperature (15 K) hysteresis loops (insets) after annealing at 700 °C. The total area of distributions is normalized to unity. Details are discussed in the text.

Encouraged by the quality of the bimodal Gaussian simulation, the procedure was extended to differently sized FePt NPs. Figure 15 shows both, the distributions of *K*_eff_ used to fit the experimental data as well as (see insets) the resulting hysteresis curves for 2.6 nm, 4.5 nm, 6.3 nm, and 10.5 nm NPs. In all cases, excellent agreement between experimental and simulated results was obtained. Common to all loops is a waist of the hysteresis loops at low external fields which is most obvious for the 6.3 nm NPs. In the simulation, this characteristic shape of the hysteresis requires a low-anisotropy component that is, accordingly, most prominent for 6.3 nm NPs in the *K*_eff_ distributions. Table 2 summarizes the results of the fitting for all NP diameters. Since a bimodal Gaussian distribution and particularly the weight of low and high *K*_eff_ is varied to match the experiments, the median of the total distribution was also determined and listed in Table 2. Note that the low anisotropy component is centered at 1.0–1.5∙10^5^ J/m^3^ for all NP batches, while the high anisotropy components peak in the range 1.0–1.9∙10^6^ J/m^3^ which is 3–6 times smaller than the bulk FePt anisotropy value. Within these two parameter ranges, all the differently waist-shaped hysteresis curves can be described by adjusting the relative weights of high and low *K*_eff_ components.

**Table 2:** Summary of the *K*_eff_ evaluation of FePt NPs batches using a Stoner–Wohlfarth approach. Diameter *d*, the median effective magnetic anisotropy constant *K*_eff_ and the peak value *K*_L_ and *K*_H_ for the two components are listed (details are given in the text).

Diameter (nm)	2.6	2.7	4.5	5.8	6.3	6.6	9.4	10.5

*K*_eff_ (MJ/m^3^)	1.71	1.14	1.93	0.56	0.92	1.57	1.81	1.69
*K*_L_ (MJ/m^3^)	0.15	0.11	0.10	0.10	0.11	0.10	0.10	0.10
*K*_H_ (MJ/m^3^)	1.40	1.03	2.40	0.75	1.50	1.90	1.90	1.80

The values of *K*_eff_ presented in Table 2 appear quite consistent. For example, neglecting the NP batches exhibiting a huge low anisotropy component (2.7 nm, 5.8 nm, and 6.3 nm), the median of *K*_eff_ is found in the range of 1.6–1.9∙10^6^ J/m^3^ after annealing at *T*_A_ = 700 °C. For these samples the experimental hysteresis loops in Figure 15 exhibit similar shapes (2.6 nm, 4.5 nm and 10.5 nm NPs). Averaging over the NP batches with larger *K*_eff_ medians yields *K*_eff_ = 1.74 MJ/m^3^. Taking into account the time window of the XMCD experiment (600 s) we made use of the expression 30*k*_B_*T*_B_ = *K*_eff_ ∙*V*_C_ to calculate blocking temperatures. We found a critical diameter of *d*_c_ = 5.2 nm for nanoparticle blocking at ambient temperature. This value is fully in-line with the experiments in Figure 13 showing superparamagnetic response at 4.5 nm, while at 5.8 nm an open hysteresis was found although the low anisotropy portion in this batch is dominant. Compared to an earlier evaluation of anisotropy of 0.7 MJ/m^3^ for slightly off-stoichiometric FePt NPs, the micelle-based NPs show an approximately 2.5 times larger *K*_eff_ [87].

#### 3.3.3 Lowering the phase transition temperature by ion irradiation

For technological applications phase transition temperatures above 600 °C are not favorable due to the high energy consumption as well as the time required to reach the L1_0_ phase. Moreover, high annealing temperatures restrict the usability of various substrates, e. g., due to degradation of the substrate or chemical reactions between the deposited particles and their support, e. g., silicide formation. Consequently, it is desirable to reduce the phase transition temperature. In the recent literature, two routes have been proposed to achieve this goal: The incorporation of a third element to function as a diffusion agent [88–89]. This approach, however, has often the undesired side effect that the pseudobinary alloys formed have a significantly reduced anisotropy compared to the corresponding pure system [90]. If the third element can be driven out, e.g., by phase separation, a huge number of vacancies and interstitials within the particles yielding lowered activation energies for diffusion can be created. Alternatively, the number of vacancies within the particles may also be strongly enhanced by ion irradiation. Previous experiments bombarding thin FePt films by He^+^ ions have clearly shown that defects formed while sputtering of atoms can be safely neglected [91].

The latter approach has been successfully applied to reduce the FePt phase transition temperature by more than 100 K [79]. In detail, 7 nm FePt alloy particles were prepared on Si(001)/SiO_2_ substrates by reverse micelles and bombarded with 350 keV He^+^ ions up to a fluence of 10^16^ ions/cm^2^ at 10**^–^**^7^ mbar and 300 K. For these conditions, SRIM simulations for a 7 nm FePt film yield an average number of displacements per FePt atom of 0.08 dpa. On the other hand, the average projected range of such He^+^ ions is found to be in the order of 1.5 µm, i.e., much larger than the particle diameter. Consequently, practically all projectiles penetrate through the NPs, produce defects there, and get stopped only deep in the substrate.

**Figure 16:**
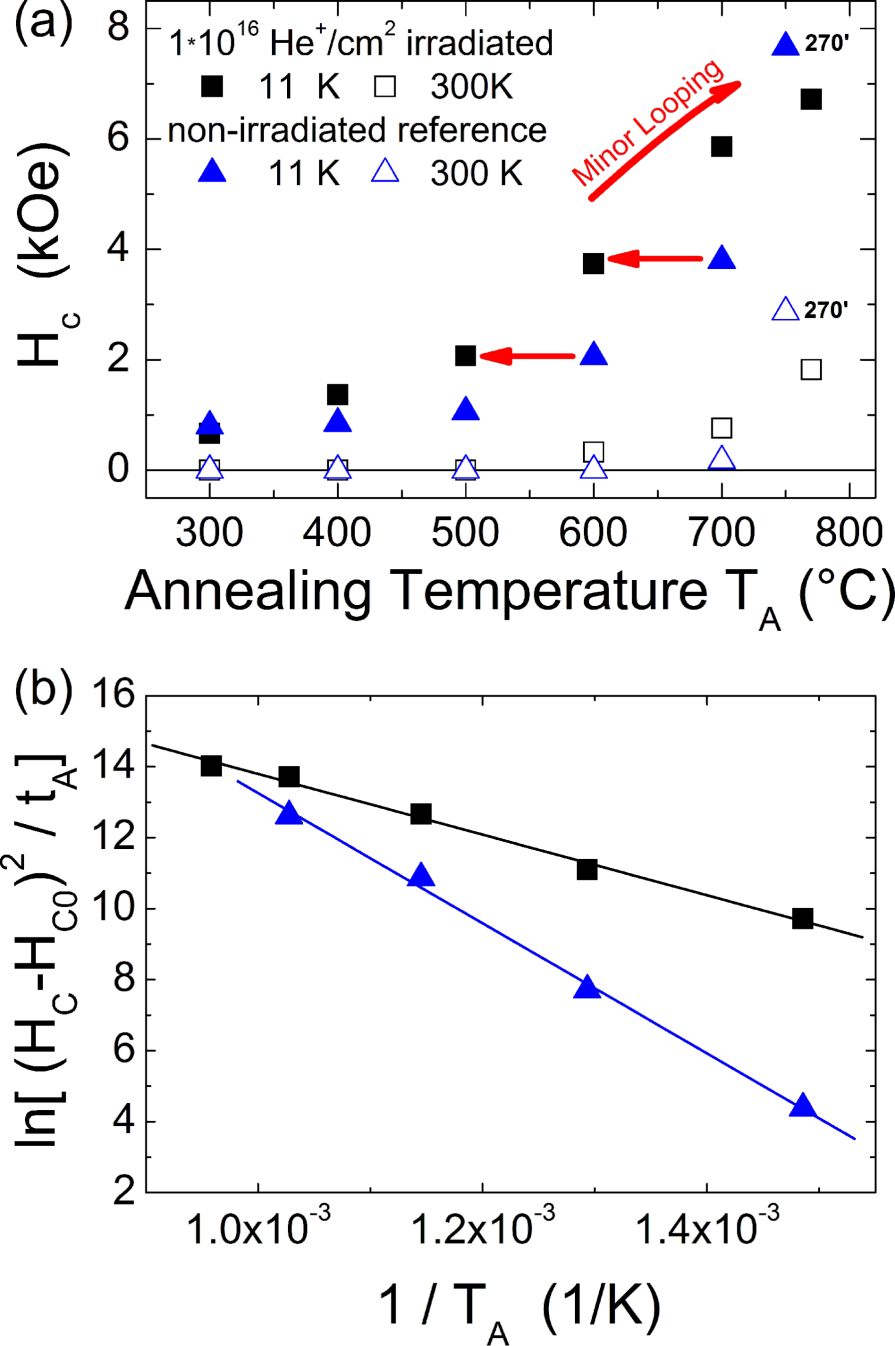
(a) Coercive fields at *T* = 11 K and *T* = 300 K as function of 30 min annealing time at temperature *T*_A_ for a He^+^ ion irradiated and a non-bombarded reference sample. The effect of a long annealing time (270 min) at 775 °C is added for comparison. (b) Arrhenius plot of normalized coercive fields with respect to the as-prepared state. From the linear fitting activation energies of 0.7 eV and 1.6 eV are derived for the irradiated and reference sample, respectively.

The influence of this irradiation process on the magnetic hysteresis loop can be compared to a non-irradiated reference sample after both have been stepwise annealed in the range 300–775 °C. The upper panel of Figure 16 shows the evolution of the coercive fields at low (11 K) and ambient temperatures as function of annealing temperature, which is maintained for 30 min at each step for both samples. Starting from small values of the coercive field related to the low-anisotropy fcc phase, increasing annealing temperatures result in a clear enhancement of *H*_C_ at 11 K in the case of the bombarded sample as opposed to the non-irradiated reference which exhibits such a significant enhancement only after annealing at 600 °C. First hysteretic behavior at ambient temperature is observed after annealing at *T*_A_ around 600 °C for the ion irradiated sample while annealing at 700 °C is necessary for the reference. The complete set of measurements of the ion bombarded sample appears shifted by more than 100 K towards lower annealing temperatures *T*_A_ as compared to the non-irradiated counterpart. For comparison, the results of long time (270 min) annealing experiments at *T*_A_ = 775 °C are included in Figure 16. Note that for huge anisotropy values (*H*_C_ > 5 kOe) the maximum experimentally available field is not sufficient to drive the sample into saturation. Thus, the coercive fields are underestimated for annealing temperatures above 700 °C.

The lower annealing temperature sufficient to form partially ordered FePt NPs after ion bombardment can be understood in terms of reduced activation energy for diffusion *E*_D_. Assuming ideal Fickian diffusion, the characteristic diffusion length λ depends on the diffusion coefficient *D* and the annealing time *t*_A_ leading to λ = (*D t*_A_)^1/2^ with *D* = *D*_0_ exp(−*E*_D_/(*k*_B_*T*_A_)) where *D*_0_ is the pre-exponential factor and *k*_B_ the Boltzmann constant. For alloys with huge magnetocrystalline anisotropy, experiments have demonstrated that the anisotropy constant has a linear dependence on the degree of chemical order *S* [92–93]. Assuming Stoner–Wohlfarth type particles showing *H*_C_ proportional to the ratio of anisotropy *K* and magnetization *M*, the coercive field is directly proportional to *S*. Using the above assumptions, it becomes possible to estimate the activation energy for diffusion *E*_D_ from the hysteresis loops of non-interacting, uniaxial, isotropically distributed FePt particles assuming (*H*_C_−*H*_C0_)/*H*_C0_ is proportional to *S* while this quantity is proportional to λ, where *H*_C0_ denotes the initial coercive field. Note these assumptions only hold for S<<1. Figure 16 (b) shows the Arrhenius plots of the quantity ((*H*_C_−*H*_C0_)/*H*_C0_)^2^/*t*_A_. From the linear fitting we derived activation energies *E*_D_ of 0.7 eV and 1.6 eV for the ion irradiated and reference sample, respectively. The observed energies are significantly smaller compared to the volume activation energy of 3.0 eV/atom reported for Pt in FePt [47]. This finding may point to additional surface diffusion which is expected to play a significant role in NPs.

#### 3.4 CoPt alloy particles

The CoPt bulk system behaves quite similar compared to FePt system [83]. L1_0_ chemical order can also be achieved in a relatively wide composition range around the equiatomic ratio. Although the anisotropy energy density of bulk CoPt *K* = 5 MJ/m^3^ [94] lies slightly below the one of FePt *K* = 7 MJ/m^3^ at low temperatures, CoPt particles should reach blocking temperatures above 300 K when the L1_0_ phase is at least partially formed. Consequently, it was worth preparing CoPt alloy particles for comparison purposes.

**Figure 17:**
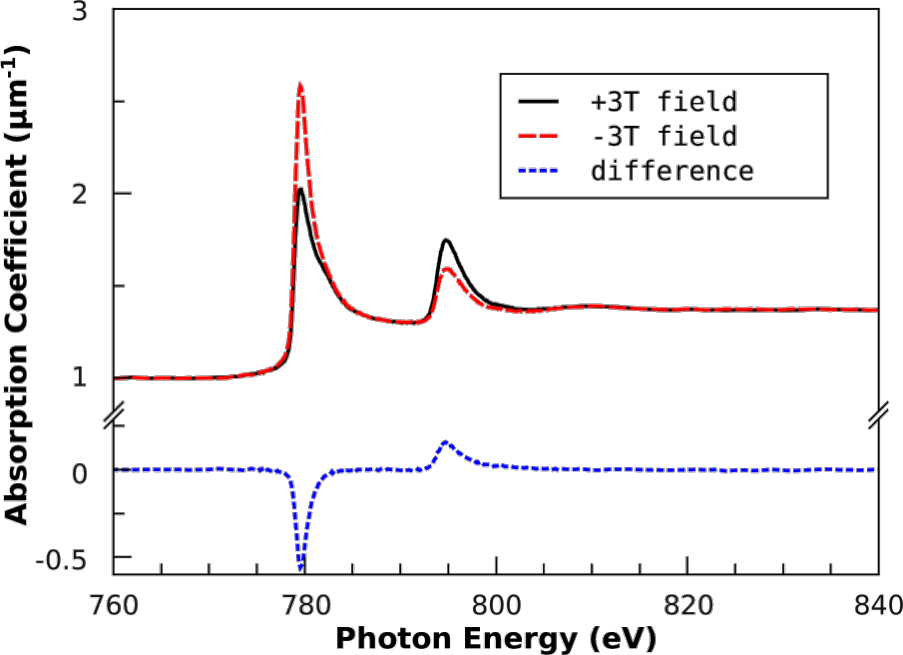
Co-L_3,2_ XA and XMCD spectra of 5.6 nm Co_56_Pt_44_ NPs after annealing at 700 °C taken at external fields of ± 3 T and *T* = 300 K. Absorption spectra are rescaled to the absolute absorption coefficient as function of energy taking into account self-absorption corrections. Details are given in the text.

CoPt NPs were prepared by reverse micelles as described above. Thus, Zeise salt as Pt precursor and CoCl_2_ were dissolved in PS(1779)-*b*-P2VP(695) reverse micelles formed in anhydrous toluene. After plasma etching, the particle height was determined as *d* = (5.6 ± 2.0) nm on Si/SiO_2_ substrates by AFM. The average particle composition was Co_56_Pt_44_ as measured by XPS. For XMCD characterization in out-of-plane geometry, the particles were reduced in hydrogen plasma at *T* = 300 °C followed by an annealing step at *T* = 700 °C for 90 min in hydrogen at *p* = 10**^–^**^3^ mbar. After cooling and pumping the plasma chamber, the sample was transferred to the XMCD chamber for magnetic characterization. Figure 17 shows Co-L_3,2_ XAS and XMCD spectra achieved for circularly polarized light at a degree of circular polarization of 93% in external fields of ± 3 T at 300 K. For each direction of external field at least two measurements were merged before further data processing. Spectra were rescaled to the linear absorption coefficient of bulk CoPt [95] at pre- and post-edge energies before self-absorption correction was applied using the method recently reported [96–97]. Finally, spin and orbital magnetic moments of Co were calculated according to the magneto-optical sum rules, see, e.g., [98] using the number of Co-3d holes of *n*_h_ = 2.628 [83].

**Figure 18:**
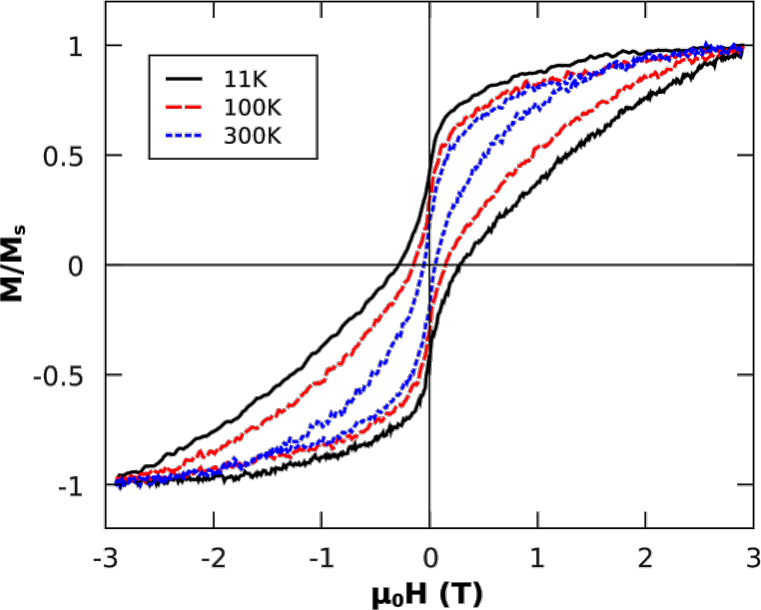
Element-specific magnetic hysteresis loops of 5.8 nm Co_56_Pt_44_ NPs taken at the Co-L_3_ edge maximum dichroic signal at *T* = 11 K, 100 K and 300 K after annealing at *T* = 700 °C for 90 min in out-of-plane geometry. Note that hysteresis loops are normalized to the magnetization at *B* = 2.9 T (maximum field).

Absorption spectra in Figure 17 clearly revealed metallic Co after particle reduction and annealing. Analysis of XMCD spectra lead to the Co spin and orbital moments listed in Table 3. Note that at *B* = 3 T, the sample is not fully saturated for all temperatures as can be seen from the hysteresis loops in Figure 18. The lack of magnetization, however, should be smallest at *T* = 11 K since all particles can be assumed to be ferromagnetic as opposed to higher temperatures where the smaller particles exhibit superparamagnetism. For the two lower temperatures, we observed constant spin and orbital moments within the error bars, while at ambient temperature a significant portion of the particles is superparamagnetic (the bump around zero external field in Figure 18) and cannot be saturated in 3 T at 300 K. The ratio of orbital and spin moments is not temperature-dependent and can be directly compared to previous reports. For an at least partially-ordered L1_0_ epitaxial CoPt film (40 nm) on MgO(001) substrates, the Co saturation spin moment has been determined to 1.76 µ_B_ (at the magic angle, see [83] for details). The spin magnetic moment of Co_56_Pt_44_ NPs of 1.6 µ_B_ (this article) is 9% smaller compared to the CoPt thin film [83] which is ascribed to an insufficient external field to reach saturation for highly anisotropic NPs.

**Table 3:** Summary of spin and orbital Co magnetic moments of 5.6 nm Co_56_Pt_44_ alloy NPs after annealing at *T* = 700 °C for 90 min. Error bars are estimated from variations of the L_3,2_ cut energy. The ratio of orbital-to-spin magnetic moments is also listed.

*T* (K)	µ_S_^eff^ (µ_B_)	µ_L_ (µ_B_)	µ_L_ / µ_S_^eff^

				11	1.60 ± 0.16	0.19 ± 0.04	0.13 ± 0.02
				100	1.56 ± 0.19	0.15 ± 0.04	0.10 ± 0.03
				300	1.26 ± 0.01	0.15 ± 0.01	0.11 ± 0.01

The anisotropy of the orbital moment turned out to be µ_L_ = 0.26 µ_B_ in CoPt(001) direction (easy axis) and µ_L_ = 0.11 µ_B_ at θ = 60° measured to the surface normal yielding µ_L_/µ_S_ ratios of 0.06-0.15 in CoPt thin films [83]. Note that the in-plane ratio is still smaller but cannot be determined due to experimental limitations. For ensembles of non-interacting particles with random distribution of anisotropy axes, however, only an integral value of the orbital moment can be measured. Recently, such experiments have been undertaken for gas-phase prepared 3 nm CoPt NPs deposited in an amorphous carbon matrix [53]. After annealing at 650 °C for 2 h, the authors observed a ratio µ_L_/µ_S_ = 0.094. Our experiments on naked Co_56_Pt_44_ NPs (5.6nm) gave an average µ_L_/µ_S_ = 0.11 and are thus in-line with the reported experiments taking into account the experimental differences and uncertainties, e.g., diameter, annealing conditions and the effect of the carbon matrix [53].

The corresponding hysteresis loops of Co_56_Pt_44_ NPs are shown in Figure 18. Similar to the results on the FePt NPs discussed above, we observed an open hysteresis loop with a coercive field µ_0_*B* around 50 mT at *T* = 300 K. The remanent magnetization, however, is strongly reduced compared to the hysteresis at *T* = 11 K showing almost 50% remanent magnetization. To get an estimate of the effective anisotropy energy density, *K*_eff_ evaluation from the hysteresis loops at *T* = 11 K was applied in-line with the model described in section 3.3.2. The median of the distribution of *K*_eff_ is found at 1.5 MJ/m^3^ which is 29% of the CoPt bulk value. Compared to FePt NPs the same trend, i.e., strongly reduced *K*_eff_ for NPs, was observed. For detailed comparison to FePt NPs, additional experiments on different sized particles in the A1 and the L1_0_ phase must be carried out.

#### 4 Long-term conservation of particles

The results discussed above have been exclusively accomplished by in situ characterization techniques. For applications, however, long-term stability at ambient conditions is a critical issue. Most magnetic systems in use tend to oxidize fast at least on the surface as shown in section 2. In other words, magnetic NPs below 10 nm always significantly oxidize when exposed to ambient conditions and thereby often lose the desired magnetic properties. Thus, encapsulation of NPs in a protective matrix or alternatively, the preparation of thin protective shells around individual particles is often necessary to prevent oxidation. Moreover, the surface functionalization of NPs is difficult and also alters the magnetism compared to naked particles. Noble metal shells provide long-term stability due to chemical inertness along with a relatively small change of the magnetism of the magnetic cores. Additionally, noble metal shells can be designed to provide distinct optical properties, e.g., by tuning the plasma frequency [99]. Last but not least the biocompatibility of Au shells has been demonstrated by many groups: Furthermore these shells provide specific molecular binding sites at the NP surface [100–101].

Recently, we have shown that Pt and FePt NPs can be covered by a thin Au shell by photochemical seeding [102–103] over macroscopic areas (10 × 5 mm^2^) in a parallel process. We therefore wished to know whether Co-based NPs could also be covered by this process. For this purpose 8 nm Co NPs were prepared on Si substrates (see section 1 for details). For photoseeding, however, the NPs were exposed to air since it is well known that Co particles form a 2**–**3 nm oxide shell when exposed to ambient conditions [104]. Thus, it was possible to study if and under what surface conditions Co-based NPs could catalyze the growth of Au shells around them.

Two different initial conditions of the 8 nm Co NPs were prepared: (i) fully oxidized Co NPs after oxygen plasma particle nucleation, and (ii) metallic Co particles after subsequent hydrogen plasma reduction. After release of the vacuum, all subsequent steps were conducted in parallel to allow direct comparison. For photoseeding a 5 mM HAuCl_4_ solution was prepared in a chemically inert optical immersion liquid (No. 1160, Cargille Laboratories). After deposition of a 20 µl droplet completely wetting the Si substrates, the solution was irradiated homogeneously with the collimated beam of an Hg lamp for 30 min (Osram; spectral emission range between 350 and 450 nm; 10.2 mW cm^−2^). After irradiation the samples were rinsed in acetone and isopropanol baths.

**Figure 19:**
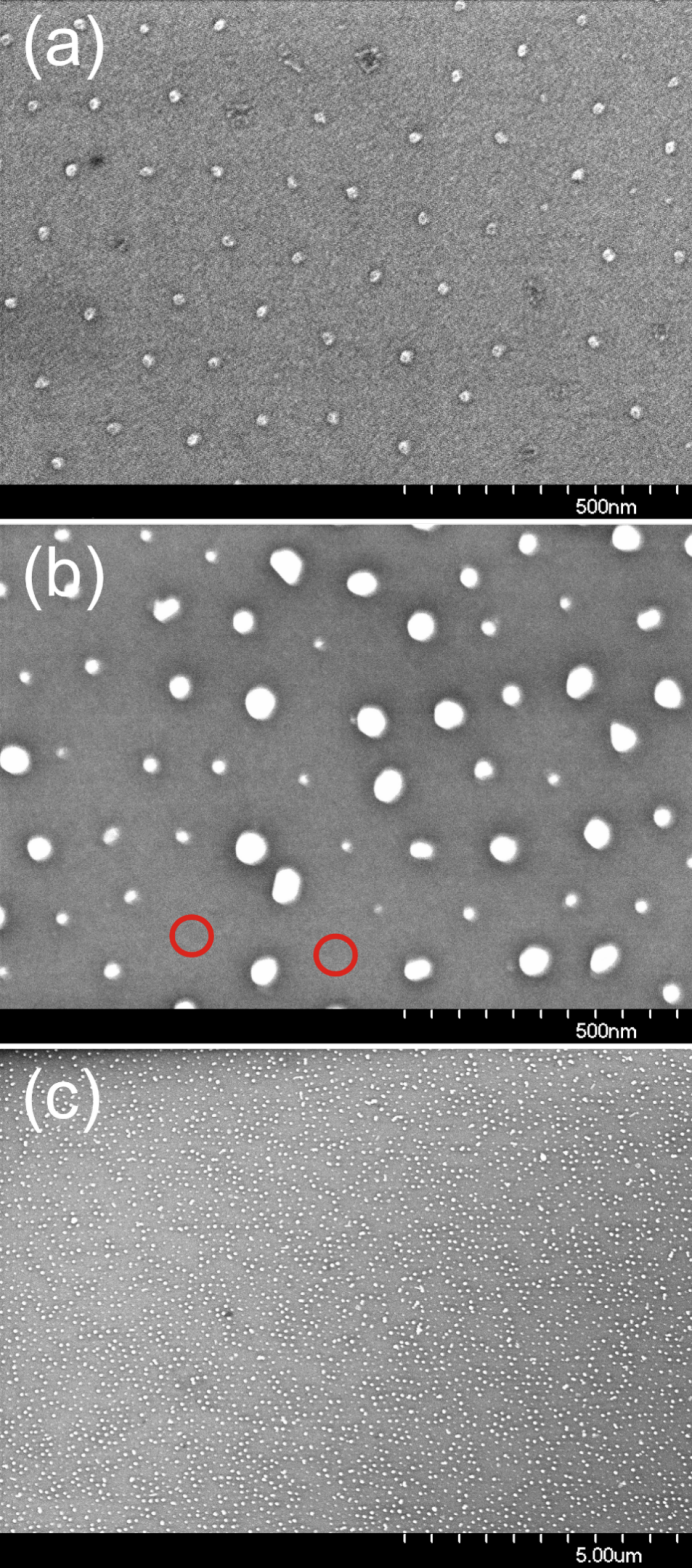
SEM images of 8 nm Co-based NP after oxygen plasma (a) and after Au photoseeding of reduced particles (b,c) at different magnifications.

The top panel of Figure 19 shows a SEM image of NPs after oxygen plasma nucleation. Note that about 10% of particles were not nucleated to single particles. In judging the growth of Au shells, however, this finding may give additional information. The photoseeding process in this fully oxidized state completely failed (not shown). In contrast, when starting from reduced NPs which form a thin Co oxide shell after about 15 min in ambient air, selective Au deposition on the NPs was possible as shown in Figure 19 (b,c). At lower magnification the homogeneity over large sample areas is clearly demonstrated. At higher magnification it is striking that (i) two different sizes of particles are observed with 10**–**15 nm and 30**–**50 nm NP diameter after Au photoseeding and (ii) some positions where NPs were expected appear void as indicated by the red circles in Figure 19 (b). The latter can be attributed to the initially incomplete particle nucleation. When several small particles were formed from a single micelle, these rapidly oxidize in air and consequently cannot catalyze the Au shell growth similar to the fully oxidized NPs discussed above.

The growth of Au shells around Co-based NPs with bimodal size distribution is harder to understand. Although the small particles in Figure 19 (b) appear to be of similar size as in Figure 19 (a), it can be concluded from the comparable SEM contrast of small and large particles that a thin Au shell has formed on all particles in Figure 19 (b). Growth rates of Au shells of less than 0.2 nm per minute for small particles and of about 1 nm per minute for large particles suggest different initial conditions. One reason might be a slightly different oxide shell thickness of the two species. Here, however, additional experiments with defined Co oxides thickness are necessary for detailed understanding of growth modes.

## Conclusion

In the present article we describe the growth, chemical and structural parameters as well as the magnetic properties of two-dimensional ordered arrays of magnetic NPs with emphasis on monatomic Co and FePt alloy NPs. The preparation route of reverse micelles, based on commercial PS-P2VP and PS-P4VP di*block*-co-polymers, leads to spherical NPs with controllable diameters *d* = 2**–**12 nm and narrow size distributions at a tunable interparticle spacing *D* = 20**–**140 nm. In all cases the ratio *D*/*d* was found being larger than 6. Micelles are successfully deposited on various planar substrate such as Si/SiO_2_, MgO, or Pt(111) textured films and, moreover, on top of AFM tips while the interparticle spacing can be additionally adjusted by the variation of dip-coating velocities. Subsequent processing by oxygen plasma forming NPs from precursor loaded reverse micelles followed by hydrogen plasma for NP reduction result in purely metallic NPs.

A host of NP systems such as Fe, Co, FePt, and CoPt NPs, were prepared and the structural, electronic as well as the magnetic properties characterized by XPS, HRTEM, SQUID-magnetometry and XMCD. For Co NPs, the formation of NPs were investigated in detail by XPS and proved the formation of Co oxide NPs after nucleation and metallic NPs after subsequent reduction in hydrogen plasma. Co NPs were successfully prepared on both, Si/SiO_2_ and Pt(111) textured films. The latter was used to compensate the diamagnetic signal of the MgO substrate at *T* = 29 K improving the application of SQUID-magnetometry and allowing direct measurement of the hysteresis loop of 8 nm Co NPs at 29 K. Moreover, DCD and IRM investigations combined in the Henkel plot deliver prove that magnetostatic interactions between particles can be neglected. Finally, Au photoseeding was accomplished on partially oxidized Co NPs extending our previous results on Pt and FePt NPs (forming a thin Pt shell). The bimodal thickness distributions of grown Au shells around Co NPs, however, still lacks a clear explanation and further experiments are required to reveal such details.

FePt alloy NPs at approximately equiatomic composition were prepared and thoroughly investigated by XMCD and HRTEM. From XMCD-hysteresis loops, the A1-L1_0_ phase transition could be tracked and compared after annealing at *T* = 700 °C for 30 min as function of FePt NP size. At *T* = 15 K hysteresis loops with a huge coercive field up to µ_0_*H* = 1 T were obtained, while at *T* = 300 K only NPs with average diameter larger than 6 nm exhibited hysteretic behavior and whilst the smaller NPs are superparamagnetic. The experimental data at *T* = 15 K could be fitted by a bimodal Gaussian size distribution of the effective magnetic anisotropy constant *K*_eff_ in a Stoner**–**Wohlfarth approach to account for low and high anisotropy distributions probably arising from (i) NPs out of the stoichiometric range in which L1_0_ order is favored and (ii) NPs forming at least partial chemical order. This fitting approach allows the determination of the median of the anisotropy distribution *K*_eff_ which was evaluated as 1.6**–**1.9∙10^6^ J/m^3^ independent of NP diameter. Such values are 3**–**4 times smaller than that of FePt bulk in the L1_0_ phase. Some NP batches, however, showed even more strongly reduced median *K*_eff_ values due to a larger amount of low anisotropy NPs. Reasons for the reduced *K*_eff_ values in NPs are elucidated by HRTEM investigations. The majority of NPs show a rather high degree of chemical order, but at the same time a variety of defects, crystallographic twins and stacking faults over different NPs were observed. Consequently, the reduced *K*_eff_ values were primarily attributed to crystalline defects.

Finally, 5.6 nm Co_56_Pt_44_ alloy NPs were produced to compare to FePt NPs. After annealing at 700 °C, we observed strong hysteretic behavior at *T* = 11 K. At ambient temperature the coercive field has been obtained to 0.5 kOe comparable to FePt NPs of similar size. The median of the *K*_eff_ was found at 1.5 MJ/m^3^ which is 29% of the CoPt bulk value.

The similarity of the magnetic behavior of FePt and CoPt leads to an important conclusion: Materials which derive their high magnetocrystalline anisotropy and, thus, their magnetic attractiveness for applications from a chemically ordered state, such as the L1_0_ in the present case, appear to exhibit strongly deteriorated magnetic properties when prepared as NPs with a monotonous decrease of, e.g., the coercive field with decreasing particle diameter. As a consequence, significantly larger NPs are required to guarantee temporal stability of their inscribed magnetization at ambient temperatures than what is hoped for on the basis of the materials bulk behavior. Part of this problem may be attributed to crystalline defects present in the NPs as demonstrated by HRTEM revealing also the existence of particles comprised of two or three sub-particles with different orientations. In such a case, considering projections of the easy axes onto the field direction immediately could explain the reduced *H*_c_ values. Additionally, the local chemical order may be disturbed in vicinity of a defect also leading to a depressed *H*_c_. It is interesting to note that in case of Co, no significant *H*_c_ depression was observed for NPs though defects are also expected for such particles. If defects are exclusively responsible for the depressed *H*_c_ values in L1_0_ NPs, the prospect of their application is definitely restricted. Recent work, however, indicated that FePt NPs may be only partially ordered due to a non-optimized annealing temperature leading to the observed *H*_c_ depression. The underlying mechanism is related to the theoretically predicted size dependence of the ordering temperature [105] with its monotonous decrease with decreasing particle diameter. As a consequence, at the most employed annealing temperatures of typically 700 °C an only partially ordered state may be stable or, in the worst case, the smallest NPs may be stable in the completely disordered fcc phase. This scenario offers a more optimistic prospect, since for smaller NPs the annealing temperature only has to be reduced, however, this must be accompanied by a correspondingly elongated annealing time to compensate for the reduced kinetics. Whether such compensation can be accomplished is not clear at the moment and the necessary experiments are under way.
